# Gray Matter Structural and Functional Alterations in Idiopathic Blepharospasm: A Multimodal Meta-Analysis of VBM and Functional Neuroimaging Studies

**DOI:** 10.3389/fneur.2022.889714

**Published:** 2022-06-06

**Authors:** Meng Zhang, Xiang Huang, Boyi Li, Huifang Shang, Jing Yang

**Affiliations:** Department of Neurology, West China Hospital, Sichuan University, Chengdu, China

**Keywords:** dystonia, blepharospasm, neuroimaging, voxel-based morphometry, meta-analysis, multimodal

## Abstract

**Background:**

Neuroimaging studies have shown gray matter structural and functional alterations in patients with idiopathic blepharospasm (iBSP) but with variations. Here we aimed to investigate the specific and common neurostructural/functional abnormalities in patients with iBSP.

**Methods:**

A systematic literature search from PubMed, Web of Science and Embase was conducted to identify relevant publications. We conducted separate meta-analysis for whole-brain voxel-based morphometry (VBM) studies and for functional imaging studies, and a multimodal meta-analysis across VBM and functional studies in iBSP, using anisotropic effect size-based signed differential mapping.

**Results:**

The structural database comprised 129 patients with iBSP and 144 healthy controls whilst the functional database included 183 patients with iBSP and 253 healthy controls. The meta-analysis of VBM studies showed increased gray matter in bilateral precentral and postcentral gyri, right supplementary motor area and bilateral paracentral lobules, while decreased gray matter in right superior and inferior parietal gyri, left inferior parietal gyrus, left inferior temporal gyrus, left fusiform gyrus and parahippocampal gyrus. The meta-analysis of functional studies revealed hyperactivity in right dorsolateral superior frontal gyrus, left thalamus and right fusiform gyrus, while hypoactivity in left temporal pole, left insula, left precentral gyrus, bilateral precuneus and paracentral lobules, right supplementary motor area and middle frontal gyrus. The multimodal meta-analysis identified conjoint anatomic and functional changes in left precentral gyrus, bilateral supplementary motor areas and paracentral lobules, right inferior occipital gyrus and fusiform gyrus.

**Conclusions:**

The patterns of conjoint and dissociated gray matter alterations identified in the meta-analysis may enhance our understanding of the pathophysiological mechanisms underlying iBSP.

## Introduction

Idiopathic blepharospasm (iBSP) is an adult-onset focal dystonia that has a peak age at onset in the fifth to sixth decade with a female predominance (1). IBSP is characterized by involuntary spasms of the orbicularis oculi and of other muscles around the eyes, which may lead to functional blindness and impair the quality of life for the patients (2). The frequently reported non-motor symptoms such as sensory symptoms, psychiatric disorders, sleep disorders and cognitive disturbances, together with typical motor symptoms constitute the clinical picture of iBSP (2, 3). Although dystonia has been linked to dysfunction of basal ganglia-thalamo-cortical pathway, recent cumulated evidence in different kinds of dystonia has pointing to other regions outside this circuit involved as well (4–6). As a common form of dystonia, the specific neural pathophysiological mechanisms underlying iBSP remain unclear.

Conventional neuroimaging studies and autopsy studies failed to find structural brain lesions in iBSP. Nevertheless, studies with advanced neuroimaging approaches have revealed structural and functional brain alterations in iBSP, and have advanced our understanding of the brain alterations in patients with iBSP. For example, the voxel-based morphometry (VBM) was used to measure the microstructural changes of brain, and functional magnetic resonance imaging (MRI) and positron emission tomography (PET) have been used to evaluate the functional changes. Using VBM, Etgen et al. reported gray matter (GM) increase in bilateral putamen and GM decrease in the left inferior parietal lobule in patients with iBSP when compared to healthy controls (7). While some VBM studies reported only GM increase in the bilateral primary sensorimotor cortex, cingulate gyrus and the bilateral precentral, left inferior occipital regions in patients with iBSP (8, 9). Alternately, one study found no GM changes in patients with iBSP (10). In regards to brain functional alterations, one study found increased activity in cerebellum while another revealed decreased activity in cerebellum (11, 12). Alternately, there are some studies didn't find altered activity in cerebellum (10, 13–15). Thus, these findings are variable and inconsistent, possibly owing to inter-study differences in sample size, sociodemographic and clinical features, imaging modalities and analytical approaches.

Although studies have examined structural or functional brain alterations in iBSP, the pattern for convergence or divergence in regional GM alterations remains unknown. Therefore, we performed separate meta-analysis to identify consistent and reliable GM structural and functional alterations in iBSP by integrating all eligible studies reporting whole-brain GM abnormalities and brain activity, separately. Then we undertook multimodal meta-analysis of GM structural and functional studies to detect converging findings that may be indicated as important brain nodes across different neuroimaging modalities in iBSP, using anisotropic effect size-based signed differential mapping (AES-SDM), a coordinate-based meta-analytic tool that has been widely applied in neuroimaging studies of neurological disorders (16). We also performed exploratory analyses to explore the potential associations between demographic and clinical variables and the identified patterns of GM alterations.

## Methods

### Literature Search and Study Selection

**Figure 1:**
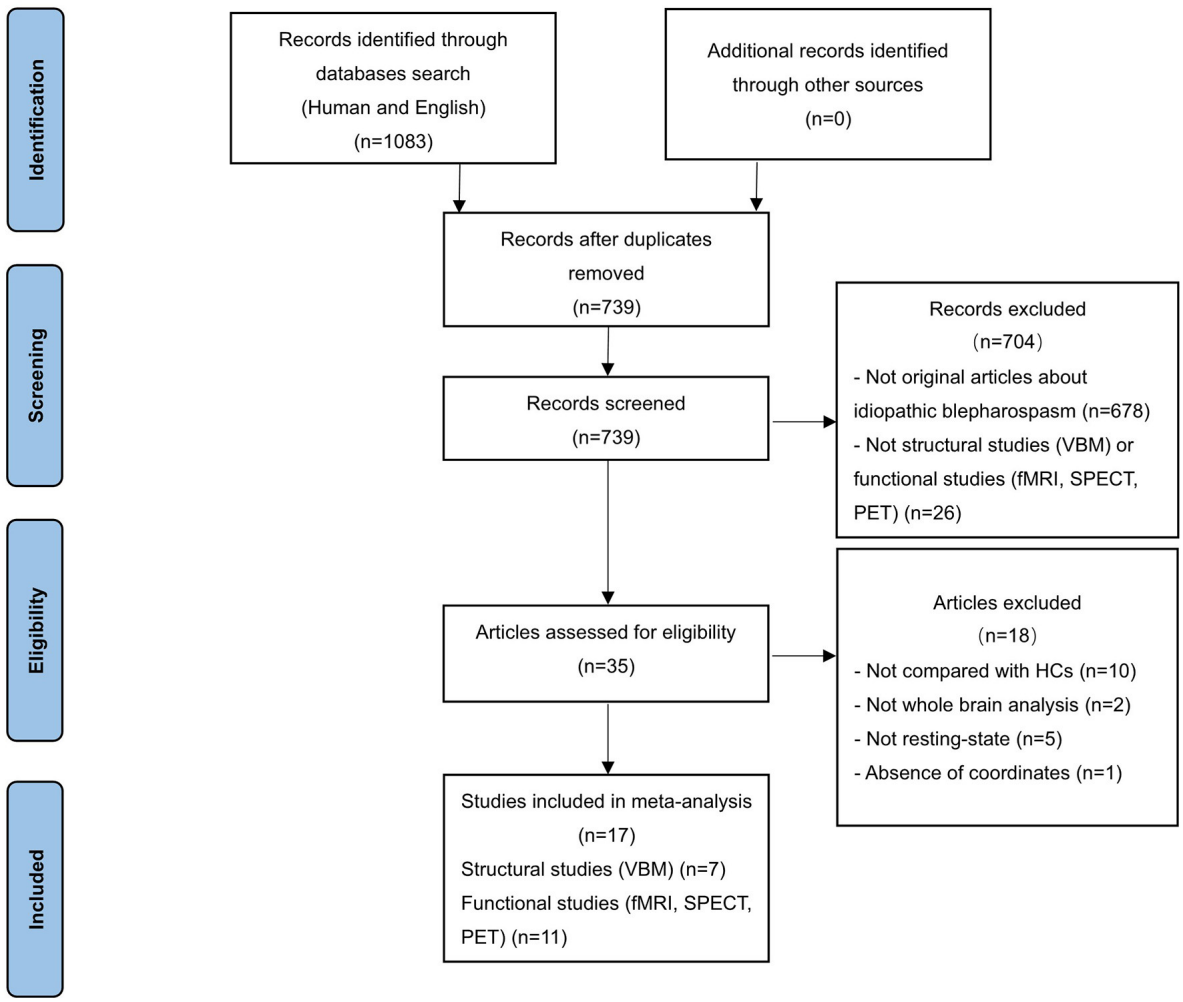
PRISMA flow diagram representing selection procedure in meta-analysis.

Systematic literature search was conducted for peer-reviewed human studies in PubMed, Embase and Web of Science published until 16 August 2021. The following English search terms were used: “blepharospasm” and (“magnetic resonance imaging” OR “MRI” OR “functional MRI” OR “fMRI” OR “positron emission tomography” OR “PET” OR “SPECT” OR “single photon emission computed tomography”). The language was restricted to English. In addition, manual searches were conducted within review articles and via the reference lists of individual studies. After duplicate removal, 739 studies were identified (Figure 1).

### Inclusion/Exclusion Criteria

Studies were considered for inclusion if they met the following criteria: (a) published in an original English paper with peer review, (b) comparison of patients with iBSP and healthy controls, (c) employed whole-brain VBM to detect GM structural changes (volume or density) or functional imaging (PET, SPECT, fMRI) to explore GM functional alterations; to minimize the heterogeneity of the functional imaging paradigms, only functional studies in the resting state were included; (d) reported whole-brain results of changes in three-dimensional coordinates (x, y, z) [Talairach or Montreal Neurological Institute (MNI)]. Studies only reporting regions of interests (ROI) findings were excluded. Studies were independently ascertained and checked by the two researchers. To achieve a high standard of reporting we have adopted “Preferred Reporting Items for Systematic Reviews and Meta-Analyses” (PRISMA) guidelines. The study selection procedures were summarized in Figure 1.

### Data Extraction

For each included study, we recorded the following demographic and clinical characteristics: sample size, sex, mean age, mean age at disease onset, disease duration, mean score of motor symptom and medication. Peak coordinates and their effect sizes (*t*-values, *z*-scores, or *p*-values) with significant differences between patients with iBSP and healthy controls in GM changes or brain functional activity were extracted from each study according to the SDM tutorial. Two authors conducted the study selection and extracted the data that needed to perform the meta-analyses independently.

### Quality Assessment

The quality of each included study was independently evaluated by two authors using a 10-point checklist adapted from previous meta-analyses (17). This assessment included the quality of the diagnostic procedures, sample size, demographic and clinical features, methods for image acquisition and analysis, and quality of reported results (see Supplementary Table S1).

### Standard Meta-Analyses of Structural and Functional Alterations

Separate voxel-based meta-analyses of regional GM and functional changes were conducted with AES-SDM package (http://www.sdmproject.com). The procedures have been described in detail elsewhere (18). This method is based on using the peak coordinates and effect sizes to recreate, for each study, an effect-size map and its variance map for the signed GM or activation differences between patients and controls, and then performing a standard random-effects variance–weighted meta-analysis in each voxel. Default AES-SDM kernel size and thresholds were used (FWHM = 20mm, voxel *p* = 0.005, peak height *Z* = 1, cluster extent = 10 voxels), as previous simulations indicated that this threshold provided an optimal balance between sensitivity and false-positive rate (16).

### Multimodal Analysis of Structural and Functional Alterations

Areas of overlapping functional and structural abnormalities between patients with iBSP and healthy controls were assessed by conjunction analysis using the multimodal meta-analysis in AES-SDM. This multimodal meta-analysis approach aims to ensure that the false-positive rate is not increased compared with that in studies of any single modality (19). We obtained a probability map of gray matter (*P*_*GM*_) and functional (*P*_*F*_) alterations to identify regions with alterations in each modality using separate meta-analysis. The multimodal analysis combined the two probabilities maps, incorporating the *P*-values to identify a union of alterations in both modalities (*U)*. The estimation of *U* is straightforward as *U* = *P*_*GM*_ + *P*_*F*_ *- P*_*GM*_ × *P*_*F*_. However, the U statistic in its raw form is overly conservative, and to reduce the imbalance between the false-positive and negative rates, *U* was adjusted according to *P* = *U*+(1-*U*) × ln(1 − *U*) (19). A more stringent probability threshold was employed for this multimodal analysis (*p* < 0.0025) than that used in unimodal meta-analyses. It should be noted that this analysis did not aim to detect correlations between structural and functional abnormalities, but to localize brain regions in which iBSP is associated with both structural and functional alterations.

### Reliability, Heterogeneity and Publish Bias Analyses

Robustness of the significant results was assessed by means of exploration of the residual heterogeneity, as well as by jackknife sensitivity analyses. Specifically, we examined the funnel plots of the peaks of maximum heterogeneity in order to check whether findings might have been driven by few or small noisy studies, or to detect any gross abnormality such as studies reporting opposite results (16). As regard to the jackknife sensitivity analysis, it was performed to test the reproducibility of results by iteratively repeating the same analyses, discarding one dataset at a time to establish whether the results remained significant (18). A result is considered replicable if identified alterations in brain area remain significant in all or most combinations of studies (18).

### Meta-Regression Analyses

The potential effect of age on group differences of regional brain structural or functional alterations were examined by a random-effects general linear meta-regression in AES-SDM. Meta-regression of other clinical features could not be performed because of limited data available. The independent variables explored by meta-regression were mean age. The dependent variable was the SDM value for VBM and regional functional meta-analyses. A more conservative threshold of *P* = 0.0005 and cluster extent = 10 voxels was used to minimize spurious findings (20).

## Results

### Included Studies and Sample Characteristics

Seventeen studies were finally included (6–15, 21–27). Among the included studies, one study reported both VBM and fMRI results (10); three fMRI studies included the same sample but reported results with different imaging measures, these results were used as from one cohort that exhibited a combination of the alterations reported in the three studies (15, 25, 27). We included seven datasets for VBM (6–10, 21, 22), with a total of 129 patients with iBSP and 144 healthy controls (see Table 1 for details), nine datasets for functional studies (10–15, 23–27), including 183 patients with iBSP and 253 healthy controls (see Table 2 for details). The demographic and clinical features of the included studies were shown in Tables 1 and 2, quality assessment of each study was done and detailed scores were shown in Supplementary Tables S2 and S3.

**Table 1:** Characteristics of included VBM studies in the meta-analysis.

**Study**	**Subjects**	**No. (F)**	**Mean age (y)**	**Onset age (y)**	**Disease duration (y)**	**Motor score of iBSP**	**Medication**	**Threshold**	**Covariate**	**Scanner**	**FWHM (mm)**	**Quality scores**
Etgen et al.	iBSP	16 (12)	67.4 ± 4.3	61.5 ± 7.1	6.5 ± 4.9	BDS = 9.5 ± 3.3 JRS-S = 1.9 ± 1.0	M	*P* < 0.001 uncorrected	Age	1.5	12	9.5
	HC	16(12)	65.3 ± 4.9									
Obermann et al.	iBSP	11(7)	52.6 ± 10.6	NA	5.5 ± 4.3	NA	M	*P* < 0.05 corrected	None	1.5	12	9.5
	HC	11(7)	52.8 ± 11.6									
Martino et al.	iBSP	25(17)	64.9 ± 7.8	NA	7.8 ± 6.2	JRS-T = 5.6 ± 1.1; JRS-S = 2.8 ±0.7; JRS-F = 2.8 ±0.5	M	*P* < 0.001 uncorrected	Age, sex, total GM volume	3.0	8	10
	HC	24(14)	63 ± 7.2									
Suzuki et al.	iBSP	32(22)	55.0 ± 6.5	49.52 ± 6.97	5.5± 4.6	JRS-S = 2.8, JRS-F = 2.8	M	*P* < 0.05 corrected	None	1.5	9	9.5
	HC	48(33)	54.4 ± 10.3									
Horovitz et al.	iBSP	14(14)	59.9 ± 6.1	51.7 ± 6.3	8.2 ± 5.5	BFM score (eye portion) = 5.61 ± 1.69	NA	*P* < 0.001 uncorrected	None	3.0	3	9.5
	HC	14(14)	58.5 ± 5.6									
Yang et al.	iBSP	18(14)	55.54 ± 8.42	51.57 ± 7.84	3.22 ± 2.01	BFM-T = 5.20 ±2.09; JRS-S = 2.60 ±0.82; JRS-F = 2.50 ±0.95; JRS-T = 5.10 ±1.68	F	*P* < 0.001 uncorrected	None	3.0	8	10
	HC	18(14)	57.27 ± 8.93									
Chirumamilla et al.	iBSP	13(8)	65 ± 6	NA	NA	NA	F	*P* < 0.05 corrected	None	3.0	8	9.5
	HC	13(5)	53 ± 7									

*iBSP, idiopathic blepharospasm; HC, healthy control; JRS, Jankovic rating scale; JRS-S, Jankovic rating scale severity; JRS-F, Jankovic rating scale Frequency; JRS-T, Jankovic rating scale total score; BDS, Blepharospasm Disability Scale; BFM, The Burke–Fahn–Marsden Dystonia Rating Scale; FWHM, full-width-at-half-maximum; NA, not applicable; F, medication free; M, on medication. Covariates, variables added when performing group comparison*.

**Table 2:** Characteristics of included functional studies in the meta-analysis.

**Study**	**Modality /analysis**	**Subjects**	**No. (F)**	**Mean age (y)**	**Onset age (y)**	**Disease duration (y)**	**Motor score of iBSP**	**Medication**	**Threshold**	**Covariate**	**Quality scores**
Hutchinson et al.	PET/ glucose metabolism	iBSP	6(NA)	63.0 ± 10.6	NA	>2 years	NA	F	*P* < 0.001	None	9
		HC	6(NA)	54.2 ± 11.4							
Kerrison et al.	PET/ glucose metabolism	iBSP	11(9)	62.9 (44–80)	NA	8(2–18)	Patients self-graded: 8 as mild, 3 as moderate	M	*P* < 0.05 uncorrected	None	10
		HC	11(9)	62.5(46–80)							
Suzuki et al.	PET/ glucose metabolism	iBSP	25(17)	52.6 ± 10.1	NA	2.9 ± 3.3	JRS-S = 0.52; JRS-F = 0.52	M	*P* < 0.05 corrected	None	9.5
		HC	38(24)	58.2 ± 7.3							
Yang et al.	fMRI/ALFF	iBSP	18(14)	55.54 ±8.42	51.57 ± 7.84	3.83± 3.93	JRS-S = 2.67 ± 0.69; JRS-F = 2.56 ± 0.86; JRS-T = 5.22 ± 1.44	F	*P* < 0.05, corrected	None	10
		HC	18(14)	57.27 ±8.93							
Zhou et al.	fMRI/ALFF	iBSP	9(7)	61.7 (52–66)	NA	2.7 ± 1.8	NA	F	*P* < 0.001 uncorrected	None	9
		HC	9(5)	52–66							
Huang et al.	fMRI/ICA	iBSP	25(17)	56.28 ± 1.89	NA	4.70 ±0.89	JRS-T = 6.36 ± 0.33	F	*P* < 0.05, corrected	None	10
		HC	25(17)	55.17 ± 1.69							
Ni et al.	fMRI/FC, ReHo, fALFF	iBSP	26(16)	56.50 ± 12.21	NA	4.19 ± 1.82	JRS-S = 3 ± 2; JRS-F =3 ± 1; JRS-T=6 ± 3	F	*P* < 0.05, corrected	Age, gender, education	10
		HC	26(18)	54.50 ± 9.59							
Wei et al.	fMRI/FC	iBSP	24(16)	49.58 ± 8.58	NA	0.92 ±0.32	JRS-S = 2.63 ± 0.82	F	*P* < 0.05, corrected	Head motion	10
		HC	24(18)	50.88 ± 8.13							
Jiang et al.	fMRI/ReHo	iBSP	24(16)	49.58 ± 8.58	NA	0.83 ±0.32	JRS-S = 2.63 ± 0.82	F	*P* < 0.05, corrected	Age	10
		HC	24(18)	50.88 ± 8.13							
Suzuki et al.	PET/ glucose metabolism	iBSP	39(27)	52.1	NA	4.9 ± 5.0	JRS-S = 2.85; JRS-F = 2.69	NA	*P* < 0.05, corrected	None	9.5
		HC	48/48(63)	55.5							
Pan et al.	fMRI/FC	iBSP	24(16)	49.58 ± 8.58	NA	0.83 ± 0.32	JRS-S = 2.63 ± 0.82	F	*P* < 0.05, corrected	Sex, age, education, head motion	10
		HC	24(18)	50.88 ± 8.13							

*iBSP, idiopathic blepharospasm; HC, healthy control; JRS, Jankovic rating scale; JRS-S, Jankovic rating scale severity; JRS-F, Jankovic rating scale Frequency; JRS-T, Jankovic rating scale total score; ALFF, amplitude of low-frequency fluctuations; fALFF, the fractional amplitude of the low-frequency fluctuation; ICA, independent component analysis; FC, functional connectivity; ReHo, regional homogeneity; NA, not applicable; F, medication free; M, on medication. Covariates, variables added when performing group comparison*.

### Individual Meta-Analytic Results

#### Regional Differences in GM Structure

**Figure 2:**
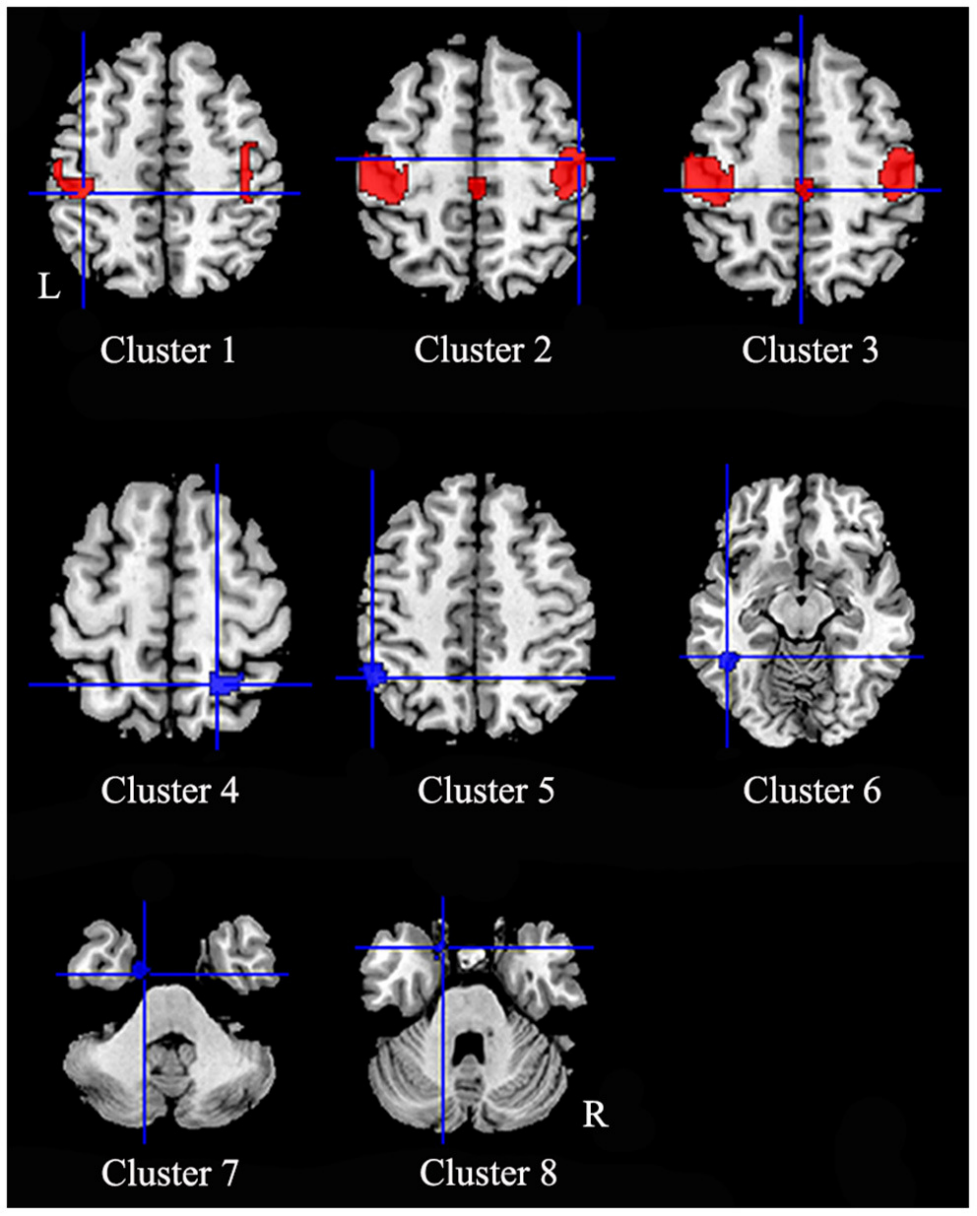
Regions of gray matter structural alterations in patients with idiopathic blepharospasm compared with healthy controls. Compared to healthy controls, patients with iBSP showed increased GM in bilateral precentral and postcentral gyri (Clusters 1, 2), right supplementary motor area and bilateral paracentral lobules (Cluster 3), while decreased GM in right superior and inferior parietal gyri (Cluster 4), left inferior parietal gyrus (Cluster 5), left inferior temporal gyrus (Cluster 6), left fusiform gyrus (Cluster 7), and parahippocampal gyrus (Cluster 8).

In comparison to healthy controls, patients with iBSP showed increased GM in bilateral precentral and postcentral gyri, right supplementary motor area (SMA) and bilateral paracentral lobules, while decreased GM in right superior and inferior parietal gyri, left inferior parietal gyrus, left inferior temporal gyrus, left fusiform and parahippocampal gyrus (Table 3; Figure 2).

**Table 3:** Clusters of gray matter structural alterations in patients with idiopathic blepharospasm compared with healthy controls.

**Regions**	**No. of voxels**	**MNI Coordinates (x, y, z)**	**SDM-Z Score**	***p*** **-value**	**Egger's test (*p*)**	**Clusters' breakdown**	**Jackknife sensitivity analysis**
**BSP > HC**							
Cluster 1	624	−40, −30, 50	1.710	<0.001	0.985	Left postcentral gyrus	7/7
						Left precentral gyrus	7/7
Cluster 2	363	48, −14, 56	1.777	<0.001	0.709	Right precentral gyrus	7/7
						Right postcentral gyrus	7/7
Cluster 3	49	0, −28, 56	1.627	0.001	0.262	Right supplementary motor area	5/7
						Bilateral paracentral lobules	5/7
**BSP < HC**							
Cluster 4	176	20, −52, 60	−1.143	0.001	0.568	Right superior parietal gyrus	6/7
						Right inferior parietal gyrus	6/7
Cluster 5	186	−56, −52, 48	−1.040	0.002	0.379	Left inferior parietal gyrus	6/7
Cluster 6	101	−46, −46, −12	−1.142	0.001	0.569	Left inferior temporal gyrus	6/7
Cluster 7	67	−14, −6, −38	−1.138	0.001	0.575	Left fusiform gyrus	6/7
Cluster 8	43	−14, 12, −30	−1.142	0.001	0.563	Left parahippocampal gyrus	6/7

*BSP, idiopathic blepharospasm; HC, healthy controls; MNI, Montreal Neurological Institute; SDM, Seed–based d Mapping*.

#### Regional Differences in GM Function

**Figure 3:**
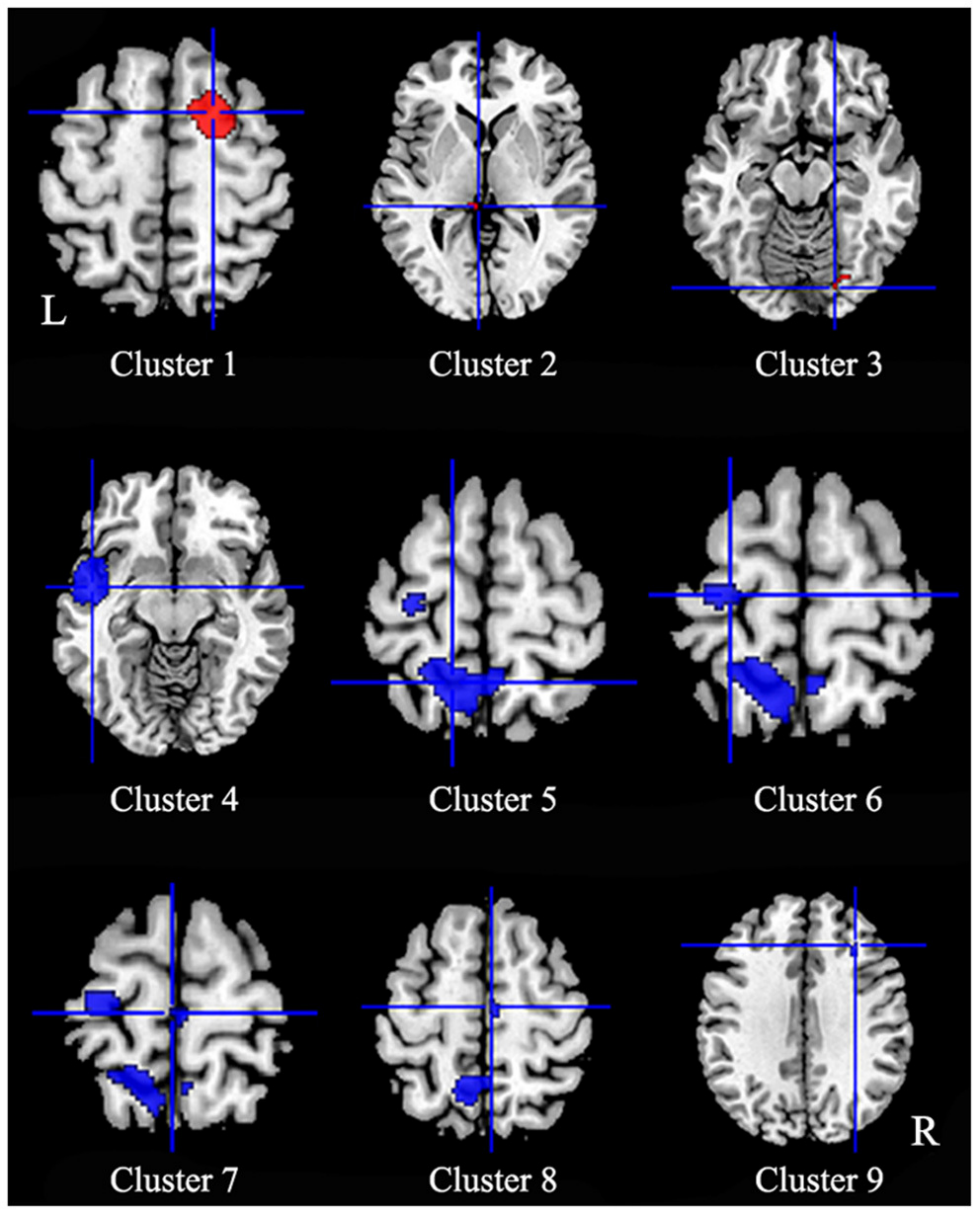
Regions of functional alterations in patients with idiopathic blepharospasm compared with healthy controls. Patients with iBSP showed increased activity compared to healthy controls in the right dorsolateral superior frontal gyrus (Cluster 1), left thalamus (Cluster 2) and right fusiform gyrus (Cluster 3), while decreased activity in left temporal pole and left insula (Cluster 4), left precentral gyrus (Cluster 6), bilateral precuneus (Cluster 5) and paracentral lobules (Cluster 7), left superior parietal gyrus, right SMA (Cluster 8), and middle frontal gyrus (Cluster 9).

Patients with iBSP showed increased activity compared to healthy controls in the right dorsolateral superior frontal gyrus, left thalamus and right fusiform gyrus, while decreased activity in left temporal pole and left insula, left precentral gyrus, bilateral precuneus and paracentral lobules, left superior parietal gyrus, right SMA and middle frontal gyrus (Table 4; Figure 3).

**Table 4:** Clusters of functional alterations in patients with idiopathic blepharospasm compared with healthy controls.

**Regions**	**No. of voxels**	**MNI** **Coordinates (x, y, z)**	**SDM-Z Score**	***p*** **-value**	**Egger's test (*p*)**	**Clusters' breakdown**	**Jackknife sensitivity analysis**
**BSP > HC**							
Cluster 1	228	20, 8, 58	1.884	<0.001	0.438	Right superior frontal gyrus, dorsolateral	7/9
Cluster 2	28	−8, −32, −2	1.753	0.001	0.170	Left thalamus	7/9
Cluster 3	19	20, −82, −14	1.610	0.002	0.268	Right fusiform gyrus	7/9
**BSP < HC**							
Cluster 4	1056	−48, −2, −10	−1.390	<0.001	0.393	Left temporal pole, superior temporal gyrus	9/9
						Left insula	9/9
						Left middle temporal gyrus	9/9
Cluster 5	431	−12, −50, 66	−1.430	<0.001	0.129	Left precuneus	9/9
						Left superior parietal gyrus	8/9
						Right precuneus	8/9
Cluster 6	73	−24, −20, 68	−1.148	0.002	0.903	Left precentral gyrus	9/9
Cluster 7	26	−2, −24, 70	−1.109	0.003	0.956	Bilateral paracentral lobules	8/9
Cluster 8	25	4, −14, 60	−1.110	0.003	0.490	Right supplementary motor area	7/9
Cluster 9	19	32, 30, 34	−1.039	0.004	0.970	Right middle frontal gyrus	5/9

*BSP, idiopathic blepharospasm; HC, healthy controls; MNI, Montreal Neurological Institute; SDM, Seed-based d Mapping*.

### Multimodal Analysis of GM Structure and Function

**Figure 4:**
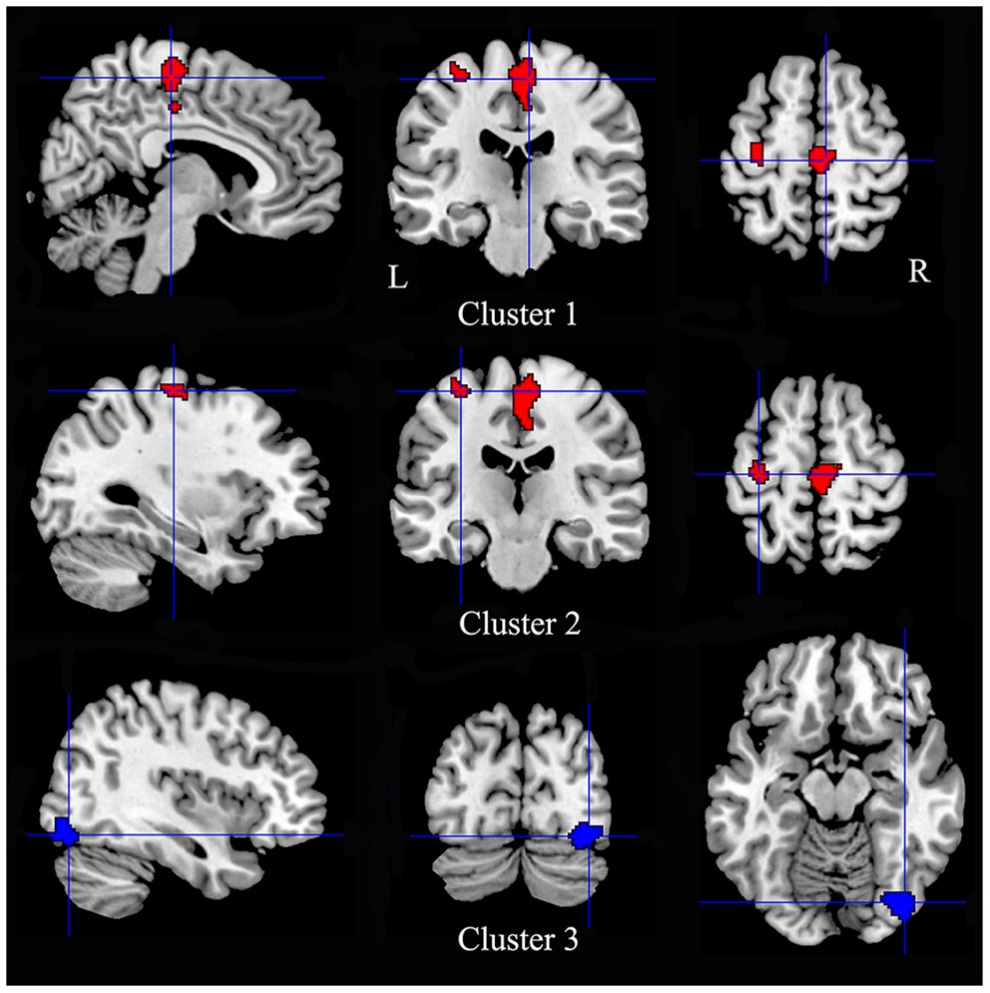
Regions showed conjoint structural and functional alterations in idiopathic blepharospasm compared with healthy controls. Patients with iBSP showed increased GM and hypoactivity relative to healthy controls in bilateral SMA and bilateral paracentral lobules (Cluster 1), and left precentral gyrus (Cluster 2). Right inferior occipital gyrus and fusiform gyrus showed GM decrease with hyperactivity (Cluster 3).

Multimodal analysis in iBSP showed increased GM and hypoactivity relative to healthy controls in bilateral SMA and bilateral paracentral lobules, and left precentral gyrus. Right inferior occipital gyrus and fusiform gyrus showed GM decrease with hyperactivity (Table 5; Figure 4).

**Table 5:** Multimodal structural and functional abnormalities in patients with idiopathic blepharospasm compared with healthy controls.

**Cluster**	**MNI**	**Voxels**	**Cluster breakdown**
**Increased GM + Hypoactivity**			
Cluster 1	6, −22, 60	309	Right supplementary motor area
			Bilateral paracentral lobules
			Left supplementary motor area
Cluster 2	−32, −20, 62	69	Left precentral gyrus
**Increased GM + Hyperactivity**			
(None)			
**Decreased GM + Hyperactivity**			
Cluster 3	38, −78, −14	184	Right inferior occipital gyrus
			Right fusiform gyrus
**Decreased GM + Hypoactivity**			
(None)			

### Meta-Regression Results

In the linear regression analysis, age did not have any significant effects on the observed between-group GM or brain activity differences.

### Reliability, Heterogeneity and Publish Bias Analyses

The whole-brain jackknife sensitivity analysis showed that the findings of GM increase in the bilateral precentral and postcentral gyri were highly replicable, being preserved in all combinations of the datasets. The GM increase in right SMA and bilateral paracentral lobules were significant in all but two combinations, all the other GM alterations were significant in all but one combination. The brain functional alterations in left temporal pole, left insula, left middle temporal gyrus, left precuneus and left precentral gyrus were significant in all combinations of the datasets. Similarly, the results in right superior frontal gyrus, left thalamus, right lingual gyrus and right SMA were significant in all but two combinations. The results of the meta-analyses thus showed high replicability and reliability in these regions. Egger's tests were non-significant, suggesting there was no evidence of publication bias for the reported clusters (Tables 3, 4). The funnel plots showed no obvious asymmetric of all significant brain regions (Supplementary Figures S1–S17).

## Discussion

To the best of our knowledge, this is the first multimodal neuroimaging meta-analysis that combines information from studies investigating whole-brain gray matter and studies investigating the functional brain on iBSP. The main findings were conjoint increased GM and hypoactivity in left precentral gyrus, bilateral SMA and paracentral lobules, and decreased GM and hyperactivity in right inferior occipital gyrus and fusiform in patients with patients with iBSP compared to controls. In addition, patients with iBSP showed decreased GM in bilateral inferior parietal and left superior parietal gyri, left inferior temporal gyri, left fusiform gyrus and left parahippocampal gyrus, while hyperactivation in right dorsolateral superior frontal gyrus, left thalamus and hypoactivity in left temporal pole and insula and bilateral precuneus. The specific and common structural and functional abnormalities revealed in the present meta-analysis may give new insight into the neuropathology of iBSP.

### Conjoint GM Structural and Functional Alterations

GM increase with hypoactivity was observed in the left precentral gyrus, bilateral SMA and paracentral lobules, regions that are functionally belong to the motor network. These results were consistent with the abnormal excitability of the primary motor cortex found in iBSP using transcranial magnetic stimulation (TMS) (28, 29). The precentral gyrus is related to the preparation and execution of movement. SMA has dense interconnections with precentral gyrus and receives major inputs from regions of the thalamus with inputs from the internal segment of globus pallidus, and a minor input from that part of the thalamus receiving inputs from cerebellum (30). SMA has a role in motor initiation, motor programming, motor planning and motor learning. In addition, increased neuronal activity in the SMA was found in response inhibition in reaction to sudden task changes (31). The hypoactivity found in bilateral SMA may be associated with losing control of unwanted involuntary movements in iBSP. Paracentral lobule is functionally connected to other frontal and parietal regions, and subserves motor functioning and spatial attention (32). The overlapping structural and functional abnormalities in these regions identified in the current study emphasized motor network changes in iBSP. The mechanistic interpretation of overlapped structural and functional altered findings was highly speculative. The findings of GM increase with hypoactivity may be the case that hypoactivity happened earlier, followed by compensatory increase in GM in the same areas, or be the case that GM increased with relatively hypo-increase of blood flow and thus as hypoactivity.

We found regions in the right inferior occipital gyrus and fusiform gyrus had GM atrophy and hyperactivity, and the left fusiform had GM atrophy. Abnormalities of structure and functional activity in these regions were also reported in other forms of dystonia (33, 34). Fusiform gyrus and inferior occipital gyrus are important components of the visual network, and are involved in the visual information-processing pathway that processes color information and facial expression perception (35). Studies have found eye blinking and interruption of visual input may influence the neuronal activity in the visual cortex (36–38). Patients with iBSP have abnormal frequency of eye blinking and excessive involuntary closure of the eyelids that may impair vision up to a functional blindness, these abnormal movements may result in altered feedback signals and an abnormal modulation of visual information and visuomotor integration. In addition, alterations of these regions may be associated with visuospatial dysfunction reported in patients with iBSP (39). The hyperactivity may be compensatory to the GM atrophy or may lead to decrease in GM by exhaustion. The underlying mechanism needs further study.

### Distinct Patterns of GM Structural and Functional Abnormalities

Several regions showed only structural or functional alterations in iBSP. The structural alterations included increased GM in bilateral postcentral gyri, decreased GM in bilateral inferior parietal gyri, left superior parietal gyrus, left inferior temporal gyrus and left parahippocampal gyrus. Although iBSP is a movement disorder, sensory symptoms including burning sensation and grittiness in the eye, dry eye and photophobia are frequently reported (3, 40). In addition, a typical sensory-motor manifestation is sensory trick that can alleviate dystonic symptoms in some maneuvers (41). The altered structural changes in bilateral postcentral gyri support deficits in sensory processing play a role in the sensorimotor integration in iBSP. Deceased GM was observed in bilateral inferior parietal gyri and left superior parietal gyrus. The parietal cortex is a critical relay station, which provides a sensorimotor interface for the control of higher-order, multimodal integration processes that are necessary to inform and guide movement execution (42). Abnormal structure, functional activity and connectivity of these parietal areas have been increasing implicated in the pathophysiology of focal dystonia (43, 44). Patients with focal hand dystonia were found to exhibit decreased connectivity of the hand region of primary sensorimotor cortex accompanied by decreased dorsal premotor and superior parietal connectivity (44, 45). Reduced GABAergic function in the inferior parietal cortex was found associated with structural alteration in patients with laryngeal dystonia, and indicated that the inferior parietal cortex may be a hub of loss of inhibition and maladaptive plasticity within the dystonia network (46). In addition, infarction lesions in the parietal cortex can induce BSP (47). The consistent structural and functional alterations in motor network, along with structural changes in postcentral gyri and parietal cortex may point to a potential pathological breakdown of the mechanisms underlying hierarchical processing and sensorimotor integration leading to dystonia movement execution.

An additional involvement of GM atrophy was found in the left inferior temporal gyrus and left parahippocampal gyrus in patients with iBSP. Inferior temporal gyrus is known to be a visual association area that receives inputs from the occipital lobe through reciprocal projection and links comprehensive information toward the prefrontal cortex for higher-order functions (48). Neuroimaging studies have suggested that the inferior temporal gyrus is involved in several cognitive processes, visual perception and multimodal sensory integration (49–51). The tractography analysis revealed extensive connections between inferior temporal gyrus and limbic areas via inferior longitudinal fasciculus, which implied the role of inferior temporal gyrus in emotion regulation (48). The parahippocampal gyrus has been scribed many functions including episodic memory, visuospatial processing and emotion processing (52). The atrophy of parahippocampal gyrus has been indicated as an early biomarker of Alzheimer's disease (53). Structural alterations in these areas may be associated with cognitive deficits and emotional disturbances reported in iBSP (3, 54).

The increased functional activities in left thalamus and right dorsolateral superior frontal gyrus, and decreased functional activities in bilateral precuneus, left temporal pole and insula were found in iBSP and not accompanied by structural alterations. Glucose hypermetabolism in the thalamus was observed in patients with iBSP, which may be related to the primary cause of compensatory mechanism of iBSP sharing the common pathophysiological mechanism to other types of focal dystonia (14). It is also reported that higher glucose hypermetabolism in the thalamus of patients with iBSP with photophobia than those without photophobia by using PET (55). The thalamus may be activated by increased sensory inputs and related to the processing of sensory signals in patients with photophobia (26). The dorsolateral superior frontal gyrus is engaged in the execution of cognitive manipulations, and was found functionally correlated with the cognitive execution network and the default mode network (DMN), especially the precuneus (56). Altered functional activity was also found in the precuneus, which is the core hub in the DMN and plays an important role in a diverse array of highly integrated functions, involving visuospatial imagery, episodic memory retrieval, self-processing and consciousness (57, 58). Dysfunction of DMN has been identified in both iBSP and other types of dystonia (27, 59). Cognitive disturbances such as executive and memory deficits reported in iBSP may be associated with alterations in DMN and cognition executive network. Further study about the impact of alterations within these networks on the cognitive disturbances in iBSP may give further understanding about this disease.

Both the temporal pole and insula are complex structures with different anatomical and functional subregions, and have wide connections with other cortical and subcortical areas. The temporal pole is associated with several high-order brain functions such as language and semantic processing, socio-emotional processing and autobiographic memory (60). Abnormalities of the temporal pole were accounted for core symptoms of various neurodegenerative diseases and socioemotional disorders (61). The insula is involved in a variety of functions including sensory processing, motor control, and emotional and cognitive functions. Alterations of these regions may be associated with the development of motor and non-motor symptoms of iBSP. Follow-up of the patients with iBSP on both clinical imaging and clinical manifestations would help clarify it.

Some limitations of this study should be noted. First, our multimodal analysis found regions showing both structural and functional alterations, but whether the relationships between structural and functional changes are causal cannot be addressed. Future studies are encouraged to detect the spatial and temporal relationships between the structure and function of these regions identified in current meta-analysis. Second, the differences in medical treatments, age of onset, disease duration, sex and age of included studies, and the difference in the preprocessing pipelines of included studies may influence the results of meta-analyses. Third, this meta-analysis was conducted based on the reported coordinates with significant differences rather than raw data from individual cases, which would affect the accuracy of the results. Fourth, we cannot determine whether these alterations are parts of the pathophysiology of iBSP or a consequence of the disease. And lastly, the GM volume or density was not specified in the results as we included studies examining GM density or volume as previous studies did (62, 63), although most (6/7) of the included VBM studies reported GM volume changes. The subgroup analysis for the VBM studies that reporting GM volume alterations were conducted, and the results were largely remained when compared with those when all seven studies were included.

## Conclusions

Our meta-analyses found that GM abnormalities in iBSP were characterized by conjoint alterations of regional structure and function mainly in the motor cortex and visual cortex, and separate GM structural changes in sensorimotor cortex and limbic lobe, and widespread altered function in the thalamus, insula, temporal pole, and regions involved in DMN and cognitive executive network. These results give us a description about underlying brain alterations that may be involved in motor and non-motor symptoms of iBSP, which may help provide new insight into the neuropathology of this disease.

## Data Availability Statement

The original contributions presented in the study are included in the article/Supplementary Material, further inquiries can be directed to the corresponding author/s.

## Author Contributions

MZ: execution, data acquisition, statistical analysis, and manuscript preparation. XH and BYL: execution, data acquisition, manuscript review, and critique. HFS: manuscript review and critique. JY: conception, organization, manuscript review and critique, and responsible for the overall content as the guarantor. All authors contributed to the article and approved the submitted version.

## Funding

This work was supported by the National Natural Science Foundation of China (grant number 81971071), the Applied Basic Research Programs of Science and Technology Department of Sichuan Province (grant number 2021YJ0447), and 1.3.5 project for disciplines of excellence-Clinical Research Incubation Project, West China Hospital, Sichuan University (grant number 2021HXFH044).

## Conflict of Interest

The authors declare that the research was conducted in the absence of any commercial or financial relationships that could be construed as a potential conflict of interest.

## Publisher's Note

All claims expressed in this article are solely those of the authors and do not necessarily represent those of their affiliated organizations, or those of the publisher, the editors and the reviewers. Any product that may be evaluated in this article, or claim that may be made by its manufacturer, is not guaranteed or endorsed by the publisher.
