# Supplementary material for: Gray Matter Structural and Functional Alterations in Idiopathic Blepharospasm: A Multimodal Meta-Analysis of VBM and Functional Neuroimaging Studies
Source: Front Neurol. 2022 Jun 6;13:889714. doi: 10.3389/fneur.2022.889714 (PMC9207395; doi:10.3389/fneur.2022.889714)
Supplement: Supplementary file 1 [file Table_1.docx]

**Supplementary Materials**

**Table and Figure legends**

**Table S1.** Quality assessment checklist.

**Table S2.** Quality assessment scores of included structural studies.

**Table S3**. Quality assessment scores of included functional studies.

**Table S4.** Clusters of gray matter structural alterations in patients with idiopathic blepharospasm compared with healthy controls (p< 0.001, uncorrected).

**Table S5**. Clusters of gray matter functional alterations in patients with idiopathic blepharospasm compared with healthy controls. (p< 0.001, uncorrected).

**Table S6.** Clusters of gray matter volume alterations in patients with idiopathic blepharospasm compared with healthy controls (p< 0.005, uncorrected).

**Figure S1.** Funnel plot of cluster 1 of increased gray matter in idiopathic blepharospasm.

**Figure S2.** Funnel plot of cluster 2 of increased gray matter in idiopathic blepharospasm.

**Figure S3.** Funnel plot of cluster 3 of increased gray matter in idiopathic blepharospasm.

**Figure S4.** Funnel plot of cluster 4 of decreased gray matter in idiopathic blepharospasm.

**Figure S5.** Funnel plot of cluster 5 of decreased gray matter in idiopathic blepharospasm.

**Figure S6.** Funnel plot of cluster 6 of decreased gray matter in idiopathic blepharospasm.

**Figure S7.** Funnel plot of cluster 7 of decreased gray matter in idiopathic blepharospasm.

**Figure S8.** Funnel plot of cluster 8 of decreased gray matter in idiopathic blepharospasm.

**Figure S9.** Funnel plot of cluster 1 of increased activity in idiopathic blepharospasm.

**Figure S10.** Funnel plot of cluster 2 of increased activity in idiopathic blepharospasm.

**Figure S11.** Funnel plot of cluster 3 of increased activity in idiopathic blepharospasm.

**Figure S12.** Funnel plot of cluster 4 of decreased activity in idiopathic blepharospasm.

**Figure S13.** Funnel plot of cluster 5 of decreased activity in idiopathic blepharospasm.

**Figure S14.** Funnel plot of cluster 6 of decreased activity in idiopathic blepharospasm.

**Figure S15.** Funnel plot of cluster 7 of decreased activity in idiopathic blepharospasm.

**Figure S16.** Funnel plot of cluster 8 of decreased activity in idiopathic blepharospasm.

**Figure S17.** Funnel plot of cluster 9 of decreased activity in idiopathic blepharospasm.

# **Table S1.** Quality assessment checklist (when criteria were partially met, 0.5 points assigned).

| **Category 1: Participants**  Score (0/0.5/1) |
| --- |
| 1. Patients were evaluated prospectively, specific diagnostic criteria were applied, and demographic data were reported.  2. Comparison participants were evaluated prospectively psychiatric and medical illnesses were excluded.  3. Important variables (e.g., age, sex, illness duration, onset, medication status, handedness) were checked either by stratification or statistically.  4. Sample size per group > 10. |
| **Category 2: Methods for image acquisition and analysis** |
| 5. Whole brain analysis was automated with no *a priori* regional selection.  6. Coordinates reported in a standard space.  7. The imaging technique used was clearly described so that it could be reproduced.  8. Measurements were clearly described so that they could be reproduced. |
| **Category 3: Results and conclusions** |
| 9. Statistical parameters for significant and important non-significant differences were provided.  10. Conclusions were consistent with the results obtained and the limitations were discussed. |
| TOTAL /10 |

# **Table S2.** Quality assessment scores of included structural studies.

| Studies | 1 | 2 | 3 | 4 | 5 | 6 | 7 | 8 | 9 | 10 | Total |
| --- | --- | --- | --- | --- | --- | --- | --- | --- | --- | --- | --- |
| Etgen et al., 2006 | 1 | 1 | 1 | 1 | 1 | 1 | 1 | 1 | 1 | 0.5 | 9.5 |
| Obermann et al., 2007 | 1 | 1 | 1 | 1 | 1 | 1 | 1 | 1 | 1 | 0.5 | 9.5 |
| Martino et al., 2011 | 1 | 1 | 1 | 1 | 1 | 1 | 1 | 1 | 1 | 1 | 10 |
| Suzuki et al., 2011 | 1 | 1 | 1 | 1 | 1 | 1 | 1 | 1 | 1 | 0.5 | 9.5 |
| Horovitz et al., 2012 | 0.5 | 1 | 1 | 1 | 1 | 1 | 1 | 1 | 1 | 1 | 9.5 |
| Yang et al., 2013 | 1 | 1 | 1 | 1 | 1 | 1 | 1 | 1 | 1 | 1 | 10 |
| Chirumamilla et al., 2019 | 0.5 | 1 | 1 | 1 | 1 | 1 | 1 | 1 | 1 | 1 | 9.5 |

#

# **Table S3**. Quality assessment scores of included functional studies.

| Studies | 1 | 2 | 3 | 4 | 5 | 6 | 7 | 8 | 9 | 10 | Total |
| --- | --- | --- | --- | --- | --- | --- | --- | --- | --- | --- | --- |
| Hutchinson et al., 2000 | 1 | 1 | 1 | 0 | 1 | 1 | 1 | 1 | 1 | 1 | 9 |
| Kerrison et al.,2003 | 1 | 1 | 1 | 1 | 1 | 1 | 1 | 1 | 1 | 1 | 10 |
| Suzuki et al.,2007 | 1 | 1 | 1 | 1 | 1 | 1 | 1 | 1 | 1 | 0.5 | 9.5 |
| Yang et al.,2013 | 1 | 1 | 1 | 1 | 1 | 1 | 1 | 1 | 1 | 1 | 10 |
| Zhou et al., 2013 | 1 | 1 | 1 | 0 | 1 | 1 | 1 | 1 | 1 | 1 | 9 |
| Huang et al., 2017 | 1 | 1 | 1 | 1 | 1 | 1 | 1 | 1 | 1 | 1 | 10 |
| Ni et al., 2017 | 1 | 1 | 1 | 1 | 1 | 1 | 1 | 1 | 1 | 1 | 10 |
| Wei et al., 2018 | 1 | 1 | 1 | 1 | 1 | 1 | 1 | 1 | 1 | 1 | 10 |
| Jiang et al., 2019 | 1 | 1 | 1 | 1 | 1 | 1 | 1 | 1 | 1 | 1 | 10 |
| Suzuki et al., 2019 | 1 | 1 | 1 | 1 | 1 | 1 | 1 | 1 | 1 | 0.5 | 9.5 |
| Pan et al., 2021 | 1 | 1 | 1 | 1 | 1 | 1 | 1 | 1 | 1 | 1 | 10 |

**Table S4.** Clusters of gray matter structural alterations in patients with idiopathic blepharospasm compared with healthy controls (p< 0.001, uncorrected).

| **Regions** | **No. of voxels** | **MNI**  **Coordinates (x, y, z)** | **SDM-Z**  **Score** | **p-value** | **Clusters’ breakdown** |
| --- | --- | --- | --- | --- | --- |
| **BSP > HC** |  |  |  |  |  |
| Cluster 1 | 34 | 48, -14, 56 | 1.777 | <0.001 | Right precentral gyrus |
| Cluster 2 | 19 | -40, -30, 50 | 1.710 | <0.001 | Left postcentral gyrus |
| **BSP < HC** |  |  |  |  |  |
| Cluster 3 | 11 | 20, -52, 60 | -1.143 | <0.001 | Right superior parietal gyrus. |
| Cluster 4 | 10 | -46, -46, -12 | -1.142 | <0.001 | Left inferior temporal gyrus |

**Table S5**. Clusters of gray matter functional alterations in patients with idiopathic blepharospasm compared with healthy controls (p< 0.001, uncorrected).

| **Regions** | **No. of voxels** | **MNI**  **Coordinates (x, y, z)** | **SDM-Z**  **Score** | **p-value** | **Clusters’ breakdown** |
| --- | --- | --- | --- | --- | --- |
| **BSP > HC** |  |  |  |  |  |
| Cluster 1 | 36 | 20, 8, 58 | 1.884 | <0.001 | Right superior frontal gyrus, dorsolateral |
| **BSP < HC** |  |  |  |  |  |
| Cluster 2 | 160 | -48, -2, -10 | -1.390 | <0.001 | Left temporal pole, superior temporal gyrus |
|  |  |  |  |  | Left middle temporal gyrus |
| Cluster 3 | 36 | -12, -50, 66 | -1.430 | <0.001 | Left precuneus |

**Table S6.** Clusters of gray matter volume alterations in patients with idiopathic blepharospasm compared with healthy controls (p< 0.005, uncorrected).

| **Regions** | **No. of voxels** | **MNI**  **Coordinates (x, y, z)** | **SDM-Z**  **Score** | **p-value** | **Clusters’ breakdown** |
| --- | --- | --- | --- | --- | --- |
| **BSP > HC** |  |  |  |  |  |
| Cluster 1 | 158 | 44, -24, 62 | 1.380 | 0.001 | Right precentral gyrus |
|  |  |  |  |  | Right postcentral gyrus |
| Cluster 2 | 66 | -6, 64, 20 | 1.255 | 0.003 | Left superior frontal gyrus |
| Cluster 3 | 47 | -28, 44, -8 | 1.253 | 0.003 | Left middle frontal gyrus |
| Cluster 4 | 25 | -18, 58, 28 | 1.256 | 0.003 | Left superior frontal gyrus |
| **BSP < HC** |  |  |  |  |  |
| Cluster 5 | 284 | 58, -22, 48 | -1.256 | 0.001 | Right supramarginal gyrus |
| Cluster 6 | 191 | 30, -50, 54 | -1.256 | 0.001 | Right superior parietal gyrus |
|  |  |  |  |  | Right inferior parietal gyrus |
| Cluster 7 | 111 | -48, -52, -8 | -1.255 | 0.001 | Left inferior temporal gyrus |
| Cluster 8 | 74 | -18, 0, -38 | -1.241 | 0.001 | Left fusiform gyrus |
| Cluster 9 | 45 | -18, 12, -26 | -1.255 | 0.001 | Left parahippocampal gyrus |

Notes: Results of the subgroup analysis for the six VBM studies that reporting GM volume alterations in patients with idiopathic blepharospasm compared with healthy controls.


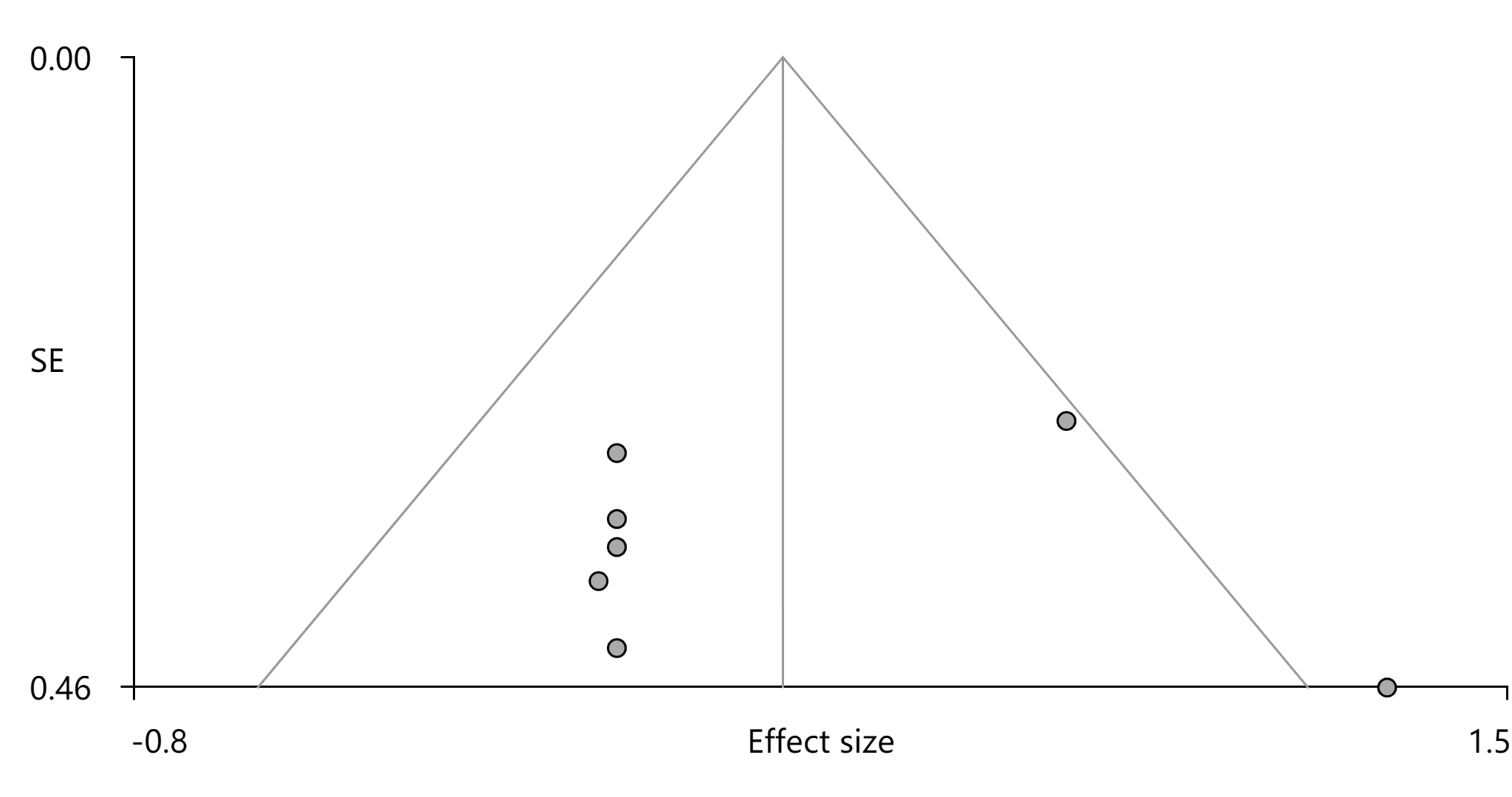
**Figure S1.** Funnel plot of cluster 1 of increased gray matter in idiopathic blepharospasm.


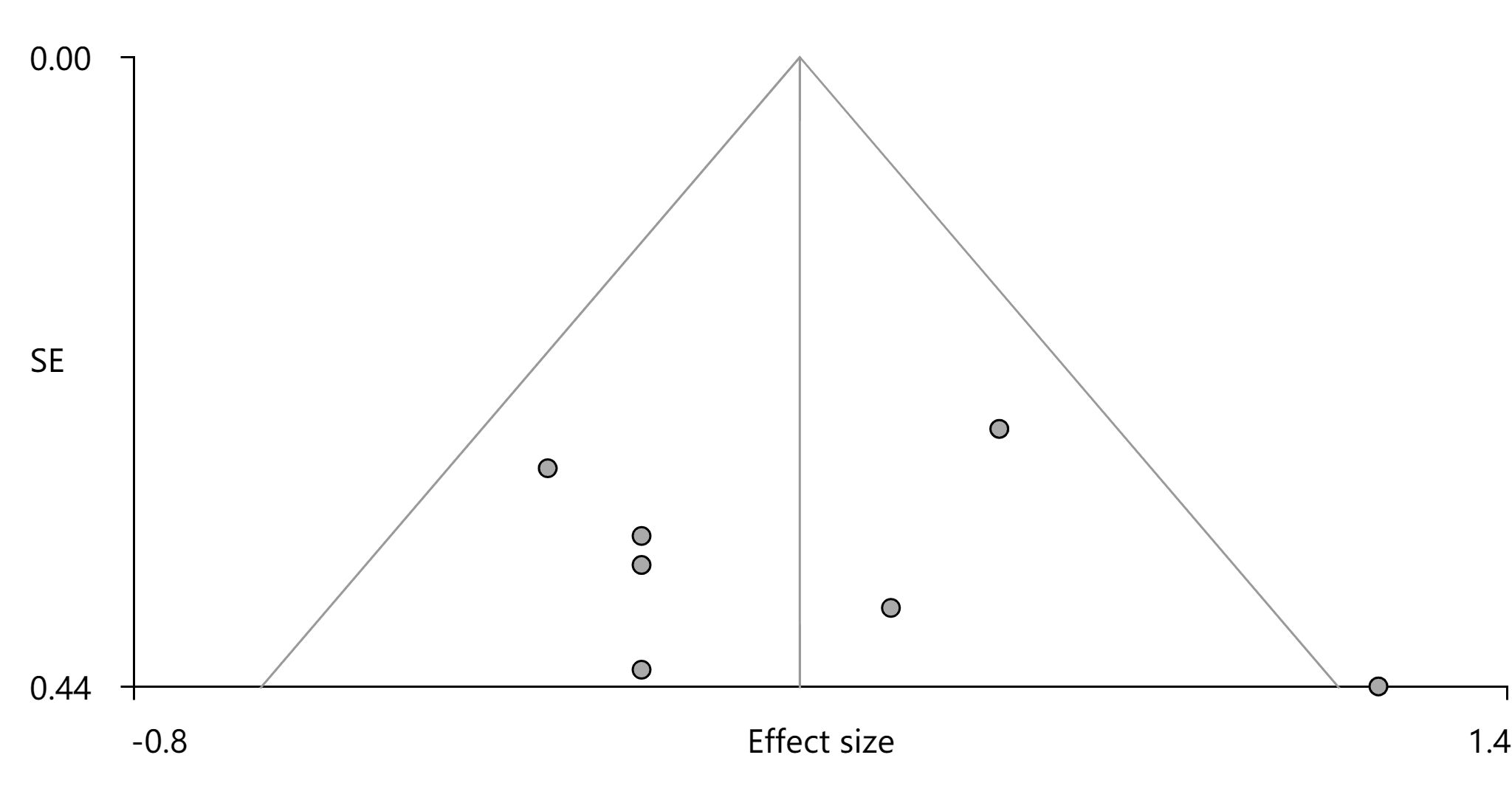


**Figure S2.** Funnel plot of cluster 2 of increased gray matter in idiopathic blepharospasm.


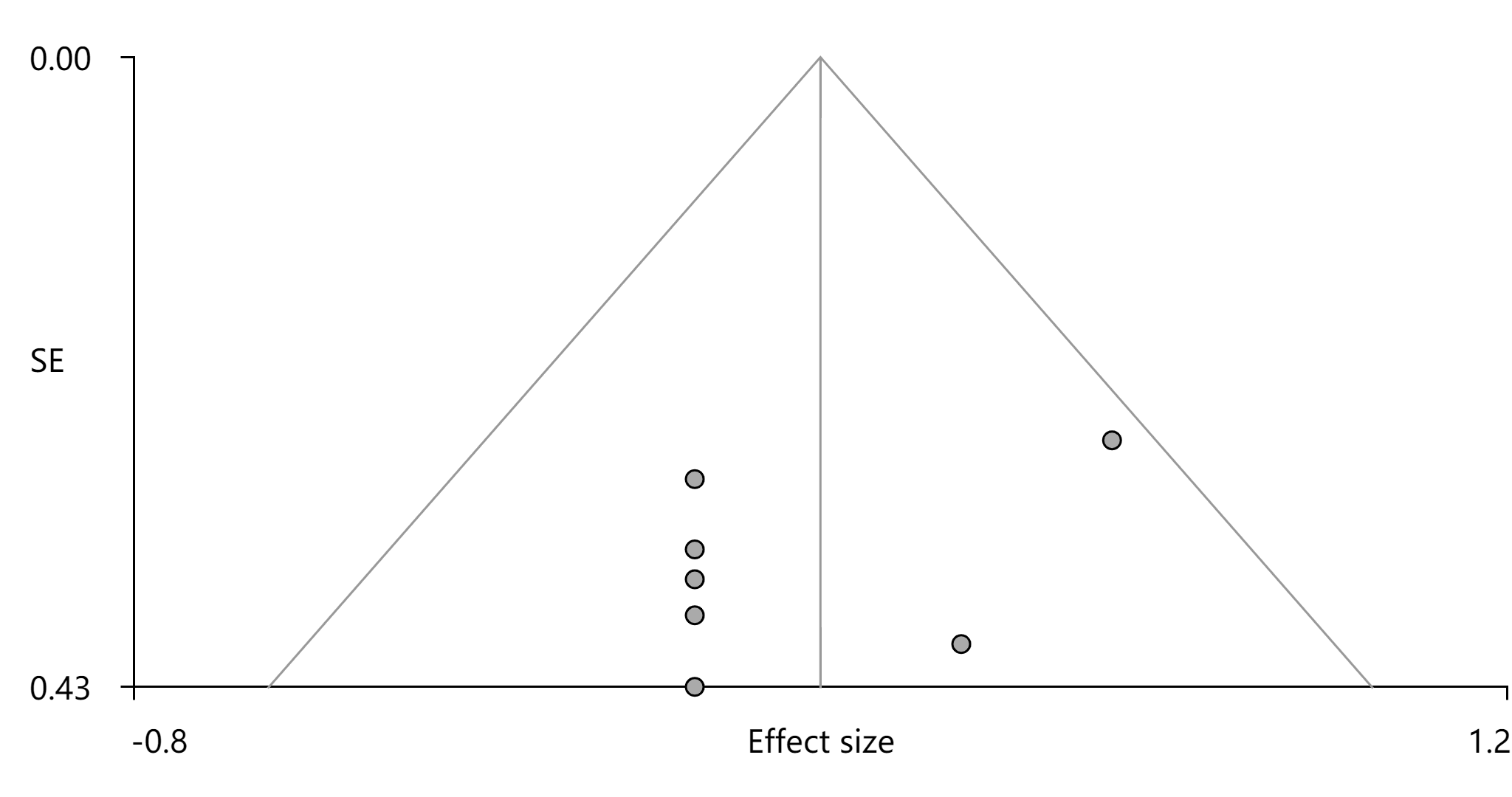


**Figure S3.** Funnel plot of cluster 3 of increased gray matter in idiopathic blepharospasm.


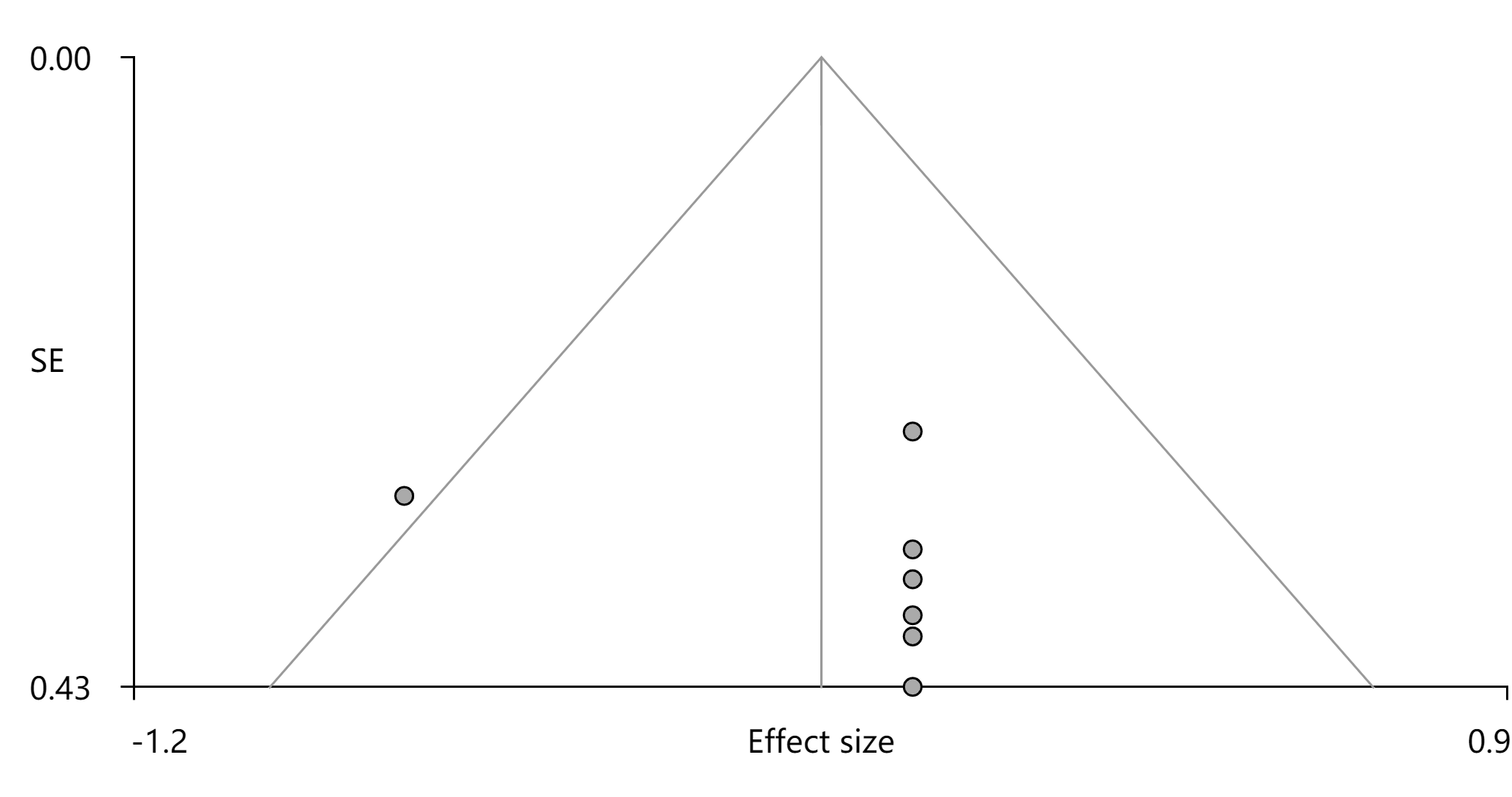


**Figure S4.** Funnel plot of cluster 4 of decreased gray matter in idiopathic blepharospasm.


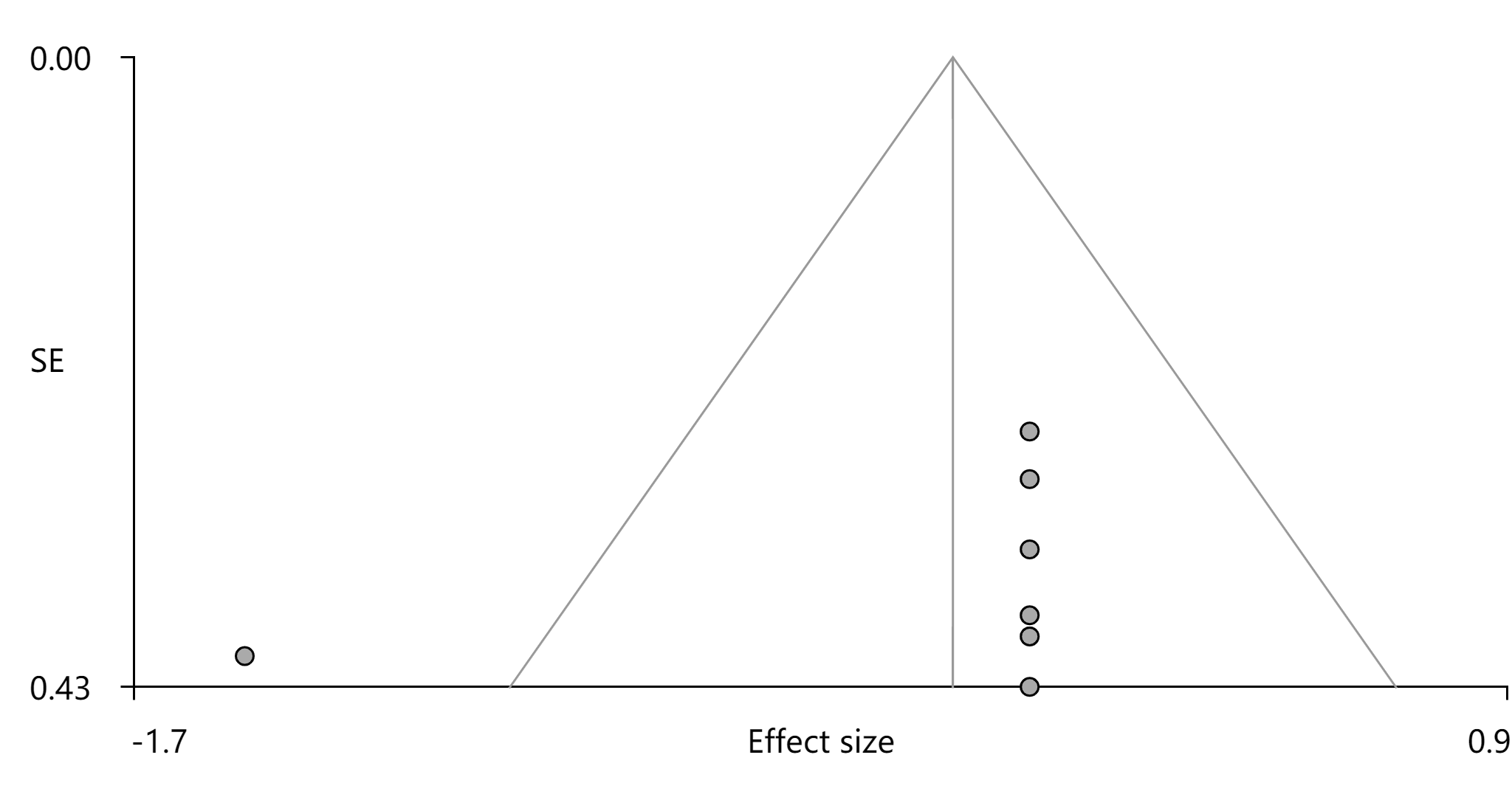


**Figure S5.** Funnel plot of cluster 5 of decreased gray matter in idiopathic blepharospasm.


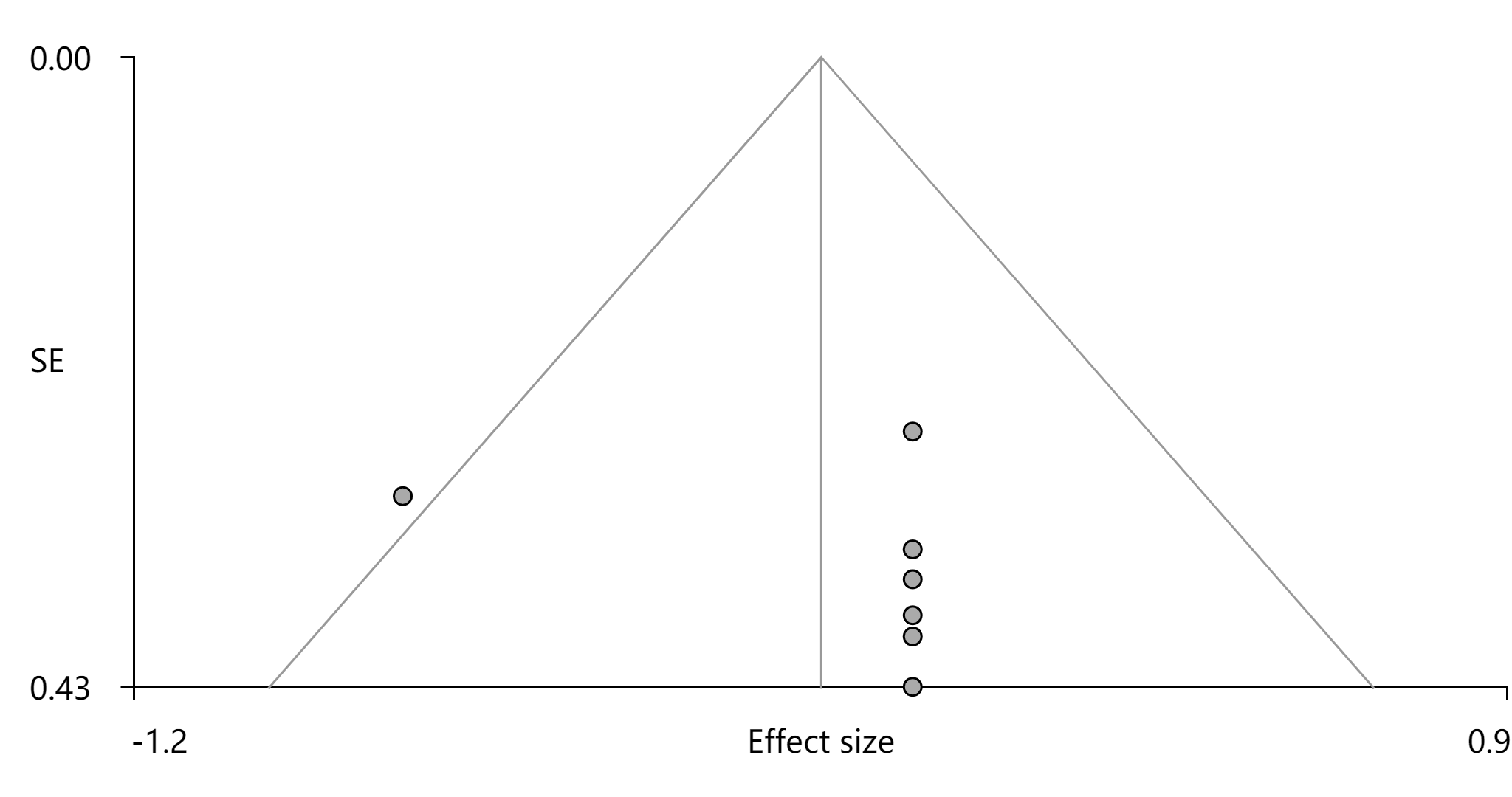
**Figure S6.** Funnel plot of cluster 6 of decreased gray matter in idiopathic blepharospasm.


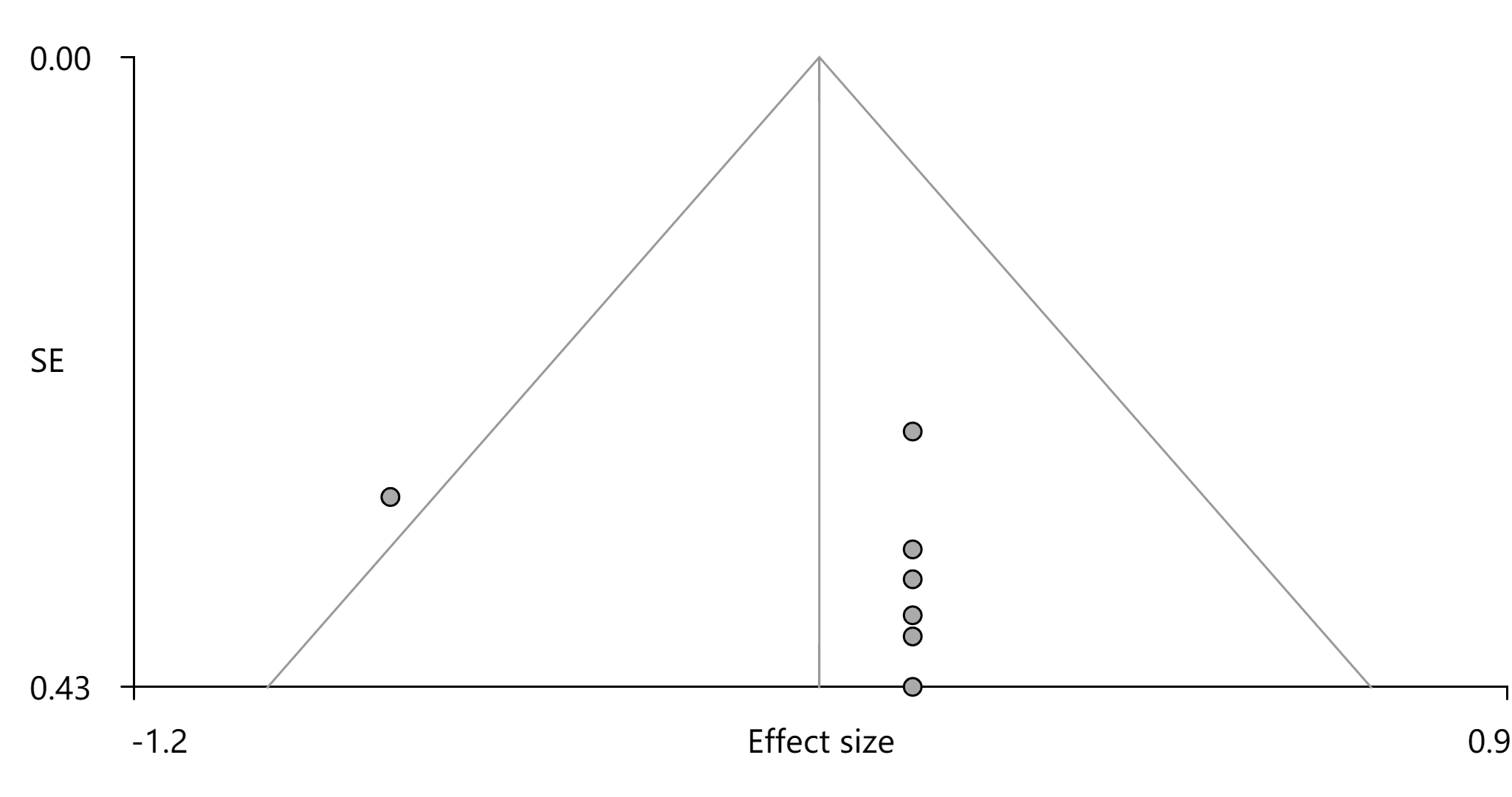


**Figure S7.** Funnel plot of cluster 7 of decreased gray matter in idiopathic blepharospasm.


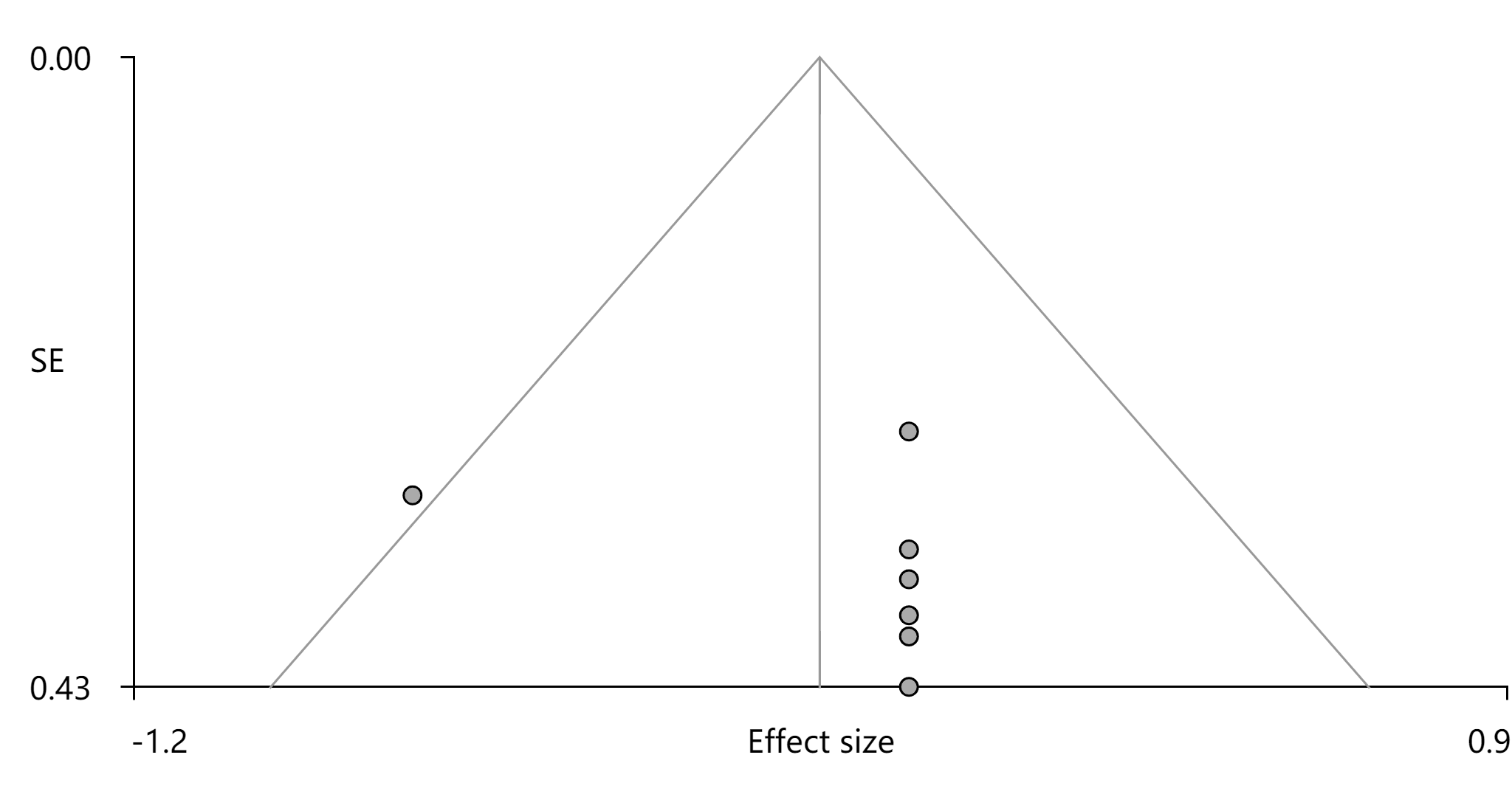


**Figure S8.** Funnel plot of cluster 8 of decreased gray matter in idiopathic blepharospasm.


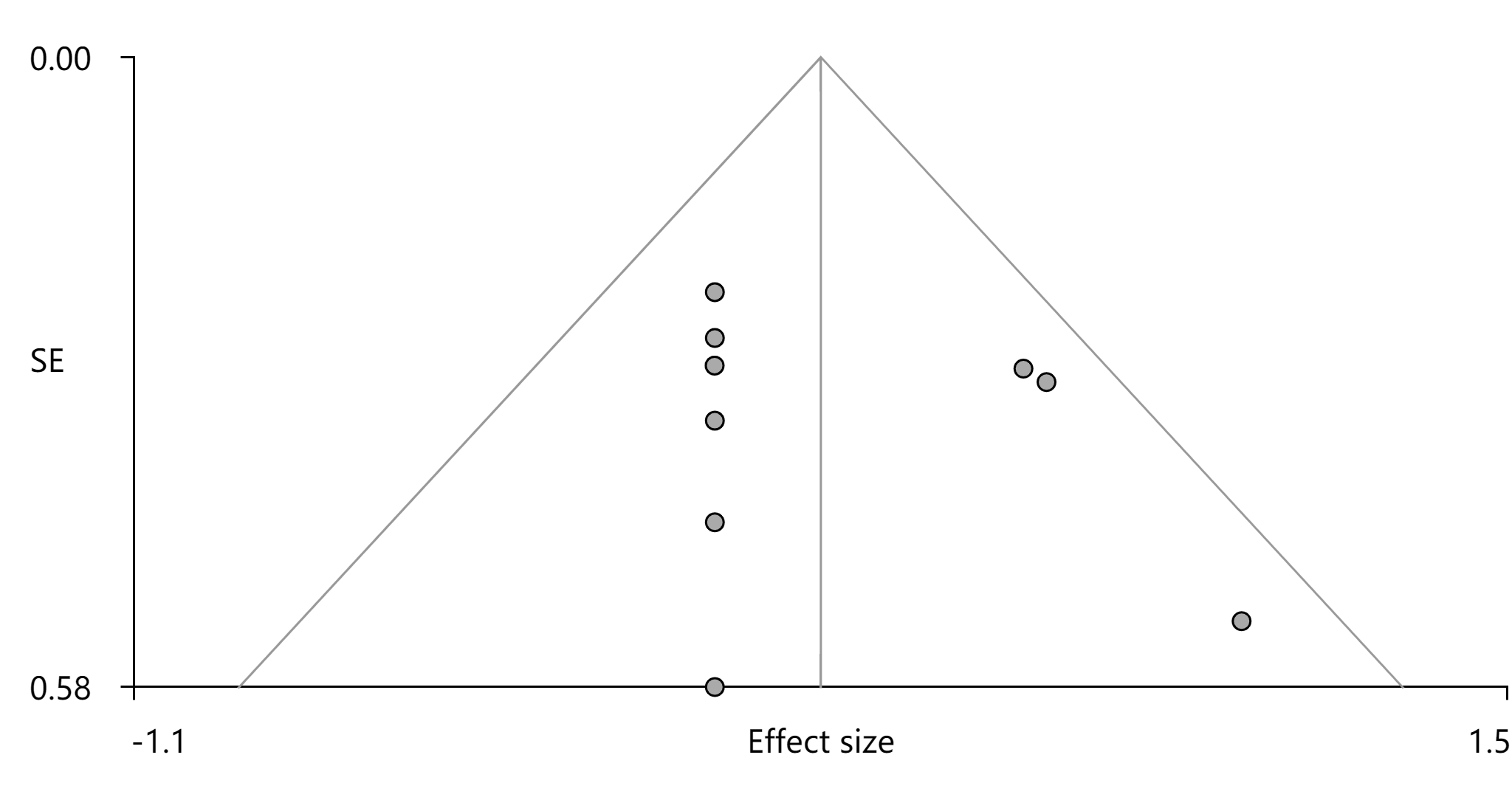


**Figure S9.** Funnel plot of cluster 1 of increased activity in idiopathic blepharospasm.


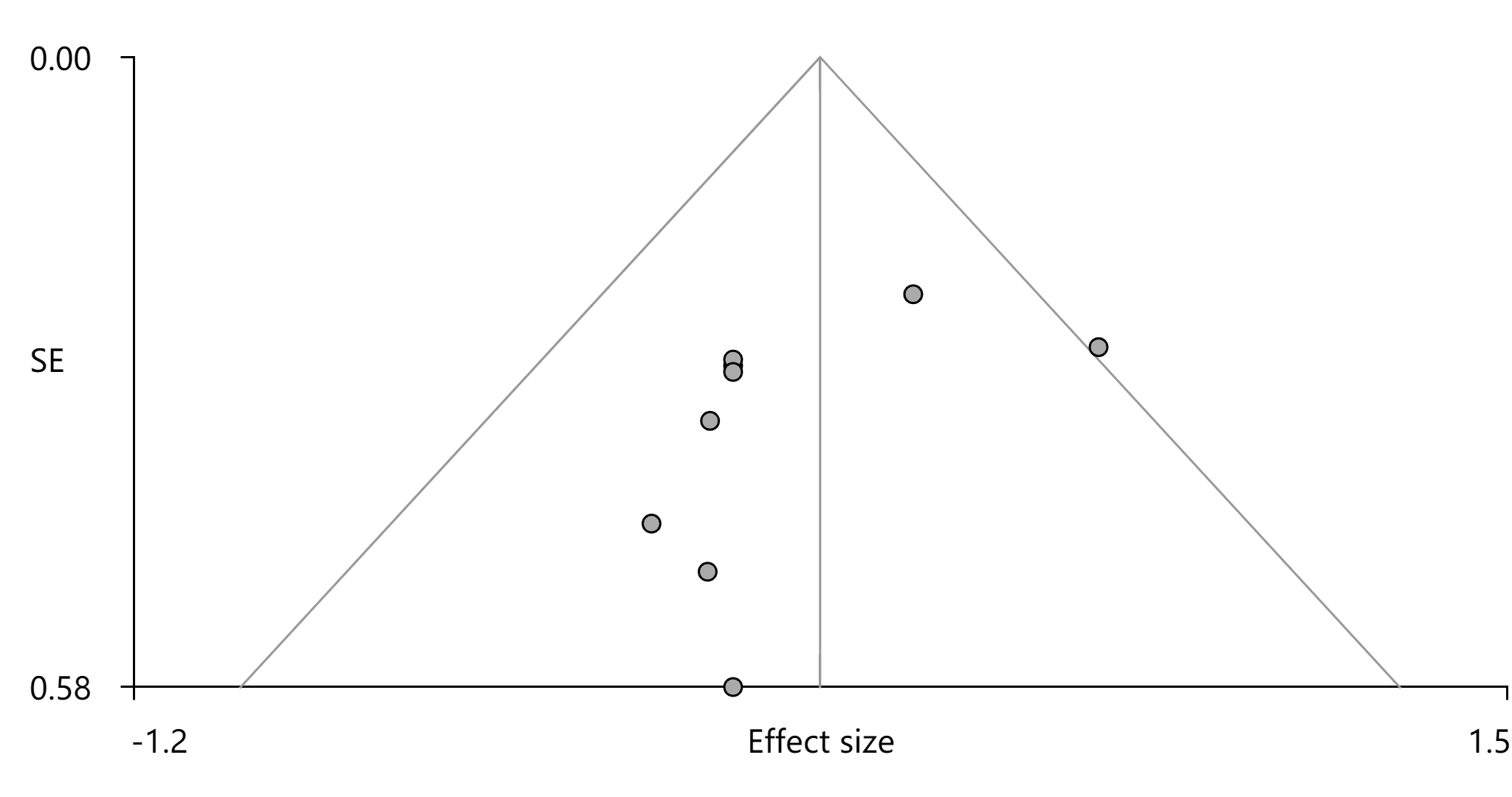


**Figure S10.** Funnel plot of cluster 2 of increased activity in idiopathic blepharospasm.


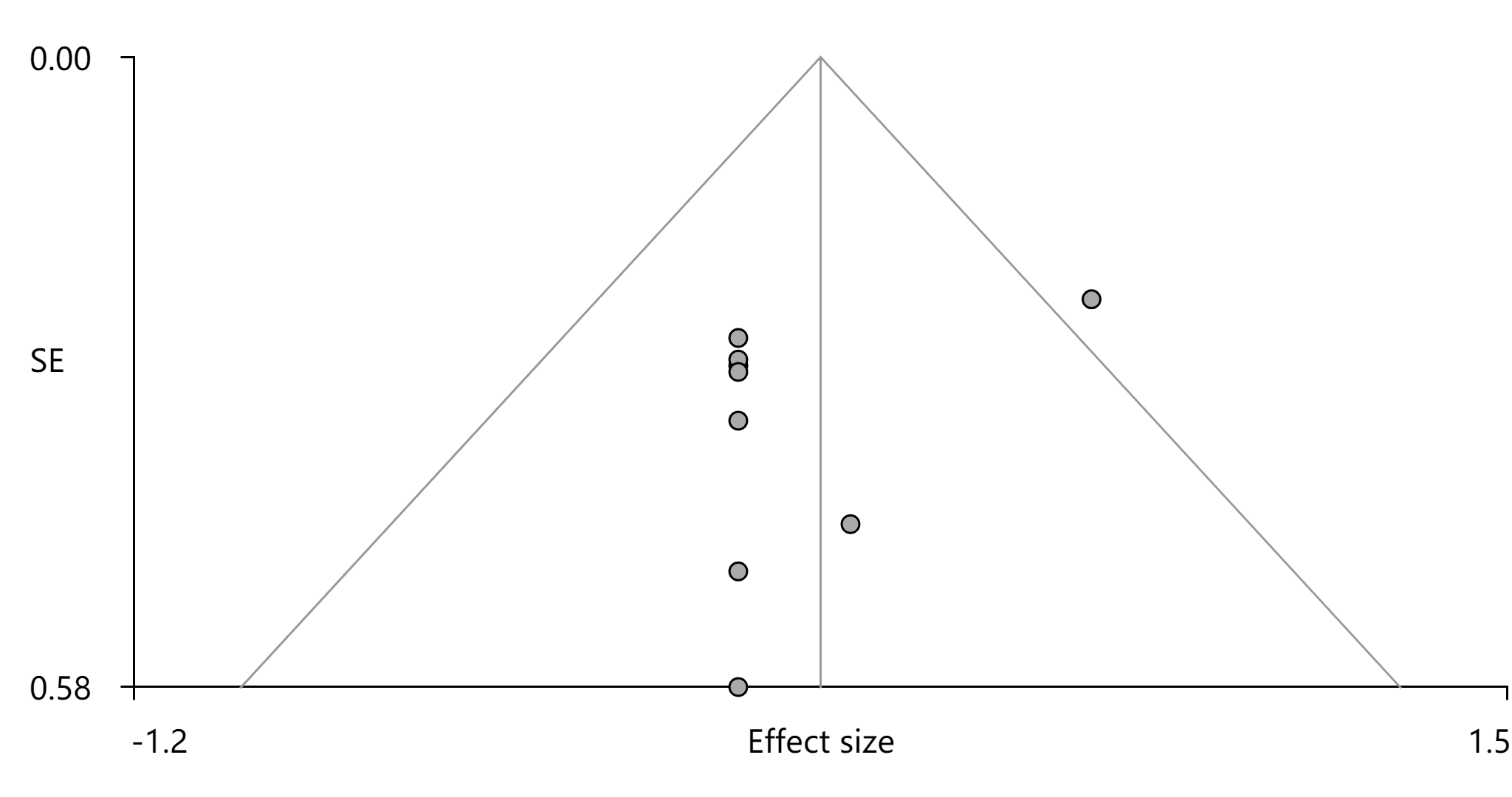


**Figure S11.** Funnel plot of cluster 3 of increased activity in idiopathic blepharospasm.


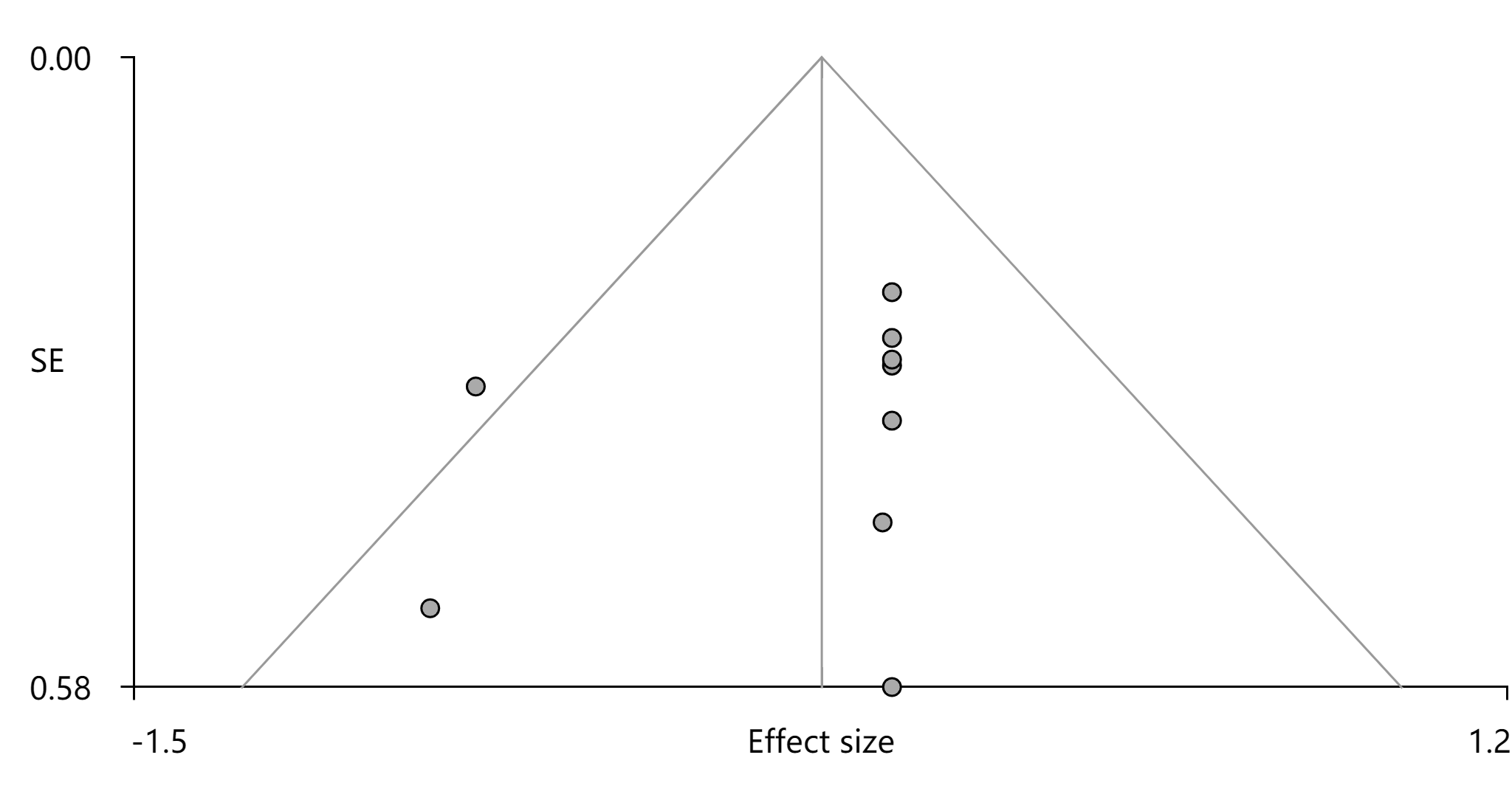


**Figure S12.** Funnel plot of cluster 4 of decreased activity in idiopathic blepharospasm.


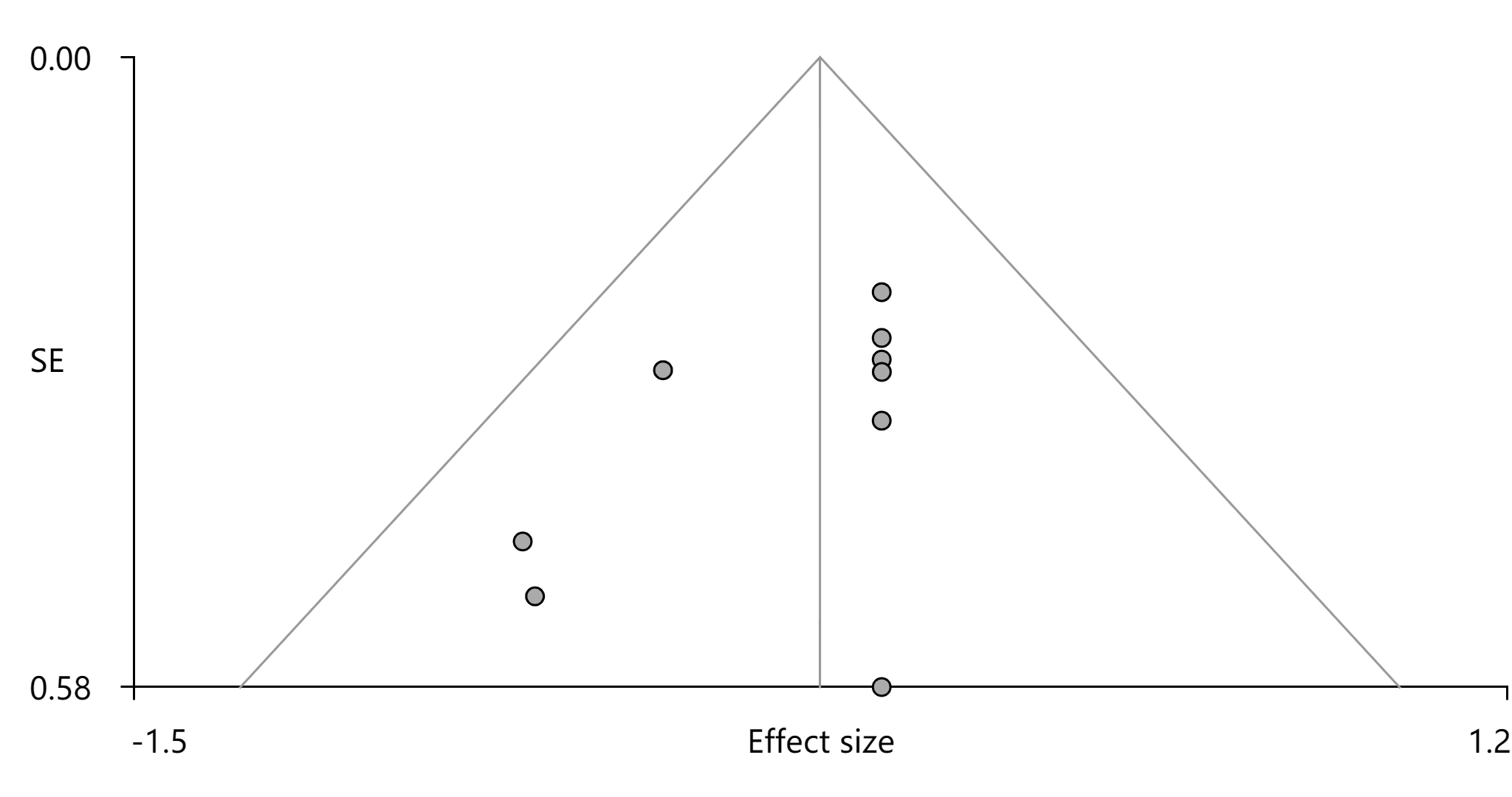


**Figure S13.** Funnel plot of cluster 5 of decreased activity in idiopathic blepharospasm.


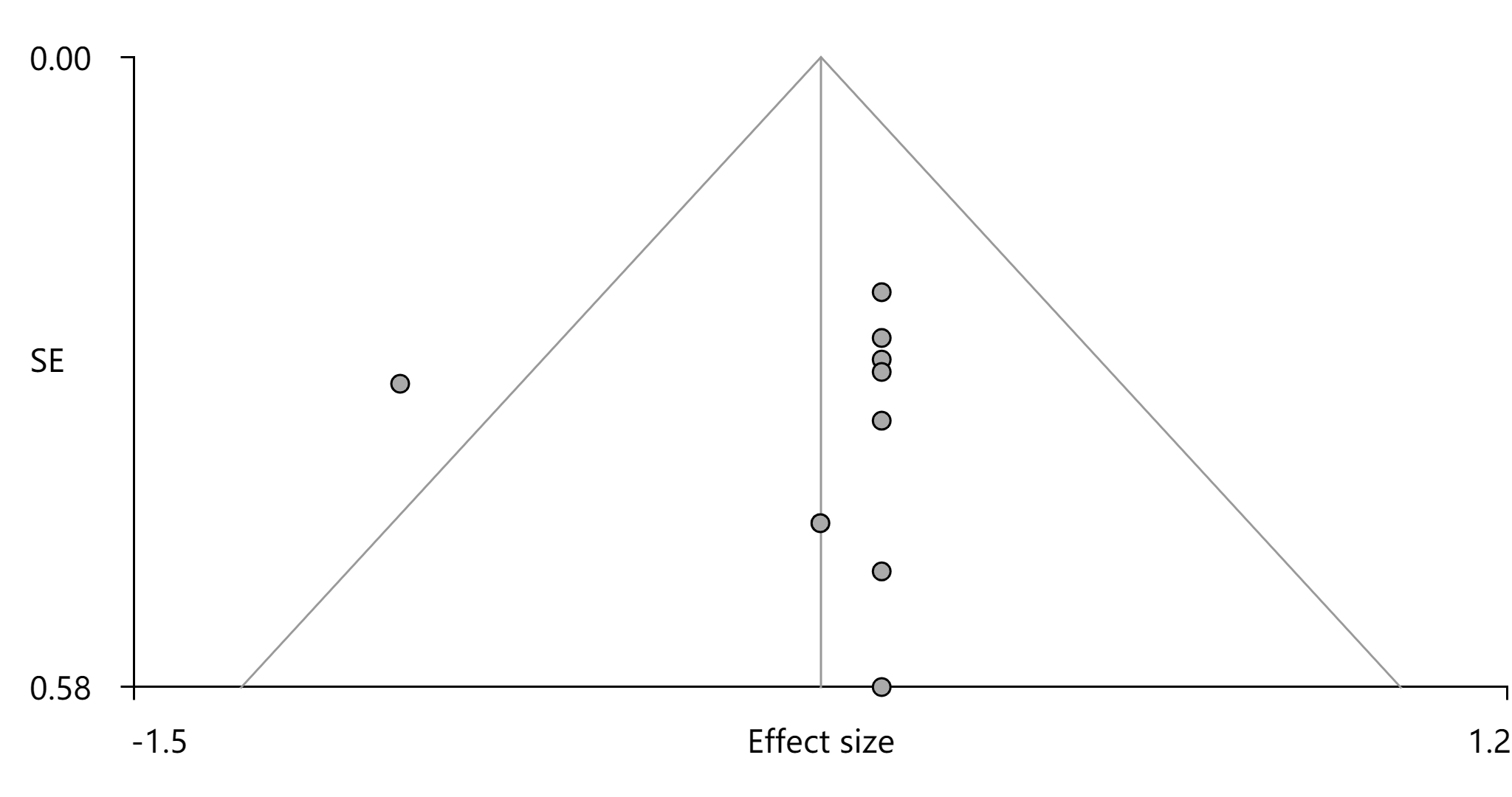


**Figure S14.** Funnel plot of cluster 6 of decreased activity in idiopathic blepharospasm.


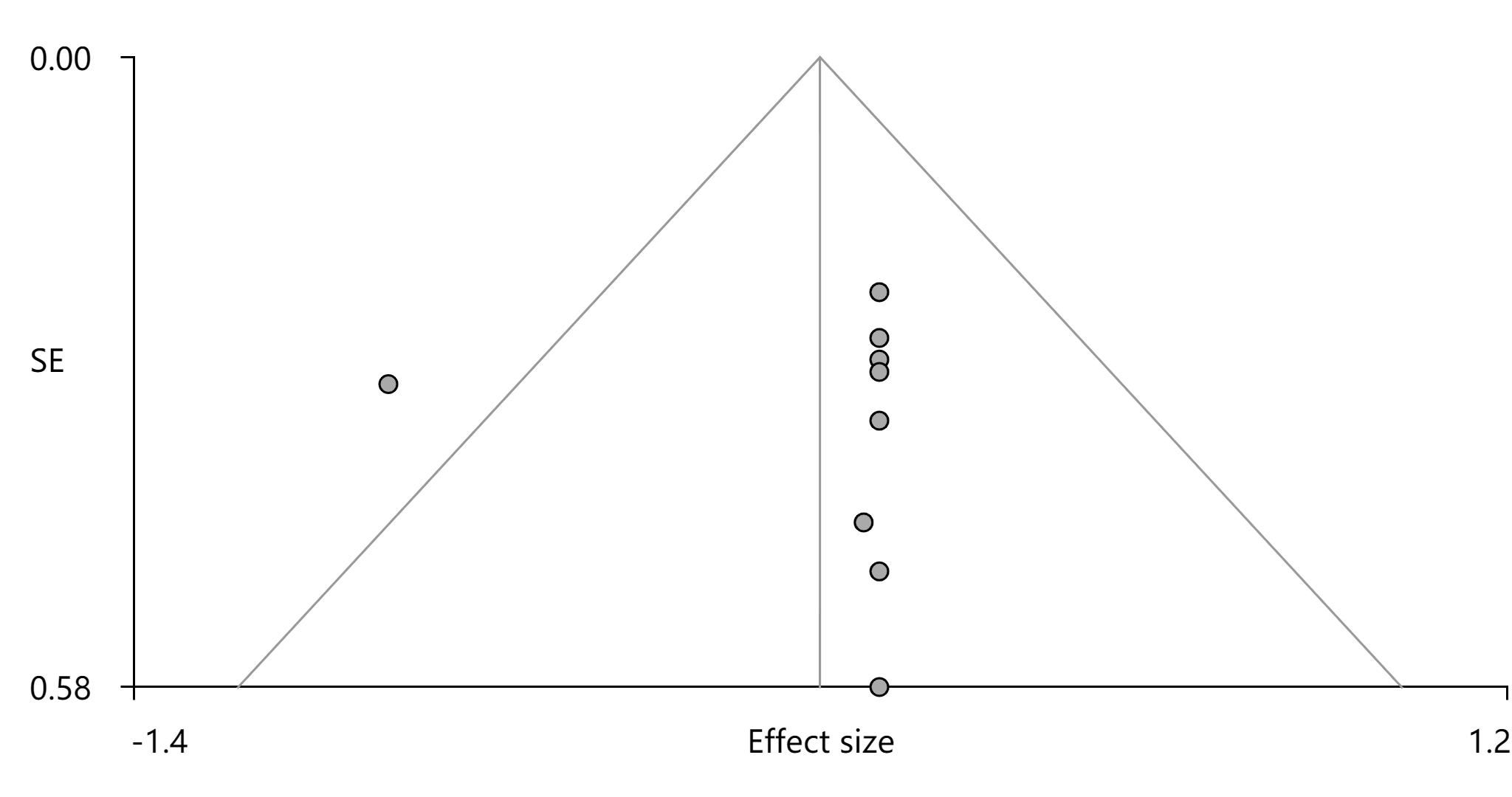


**Figure S15.** Funnel plot of cluster 7 of decreased activity in idiopathic blepharospasm.


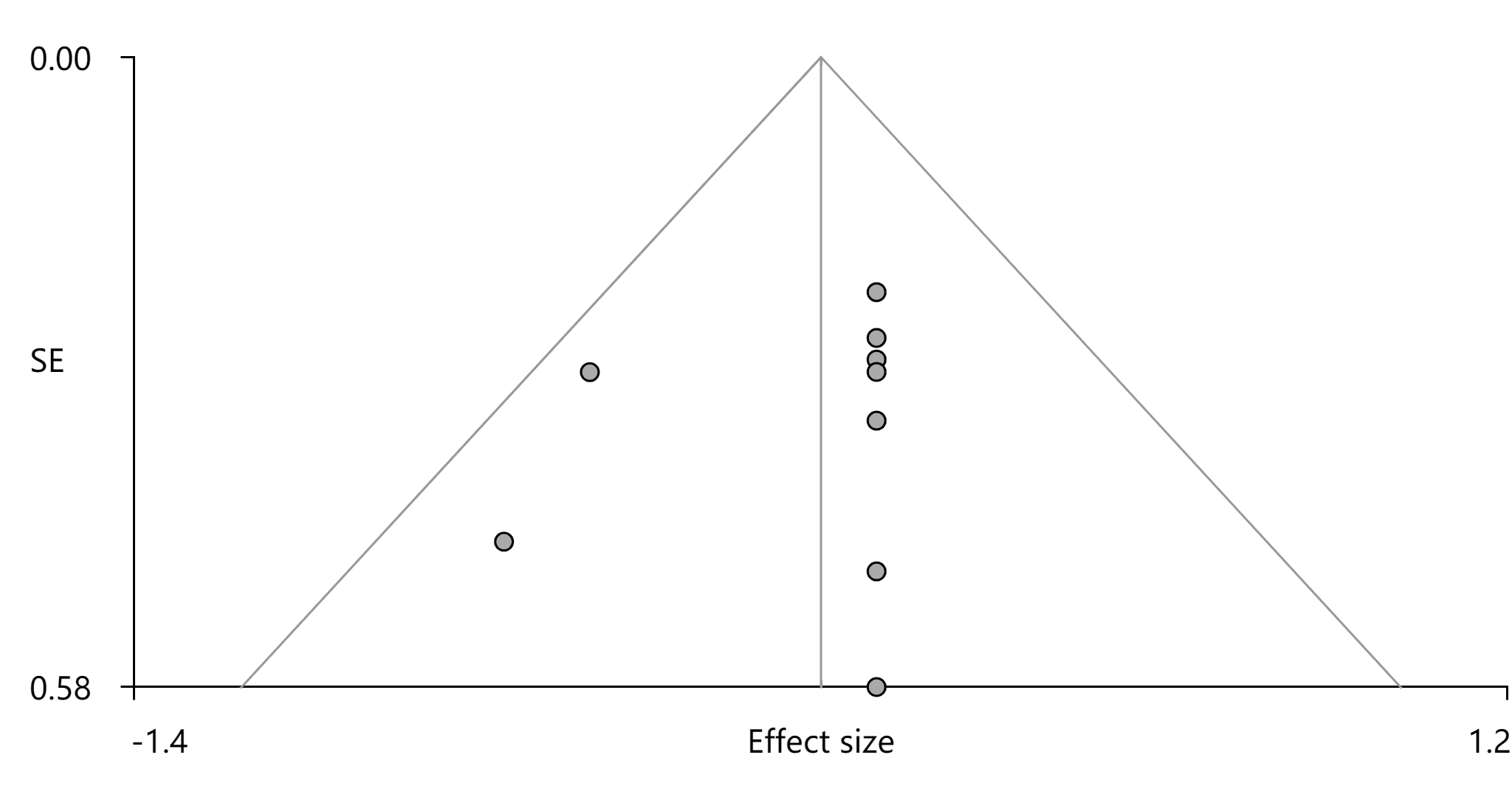


**Figure S16.** Funnel plot of cluster 8 of decreased activity in idiopathic blepharospasm.


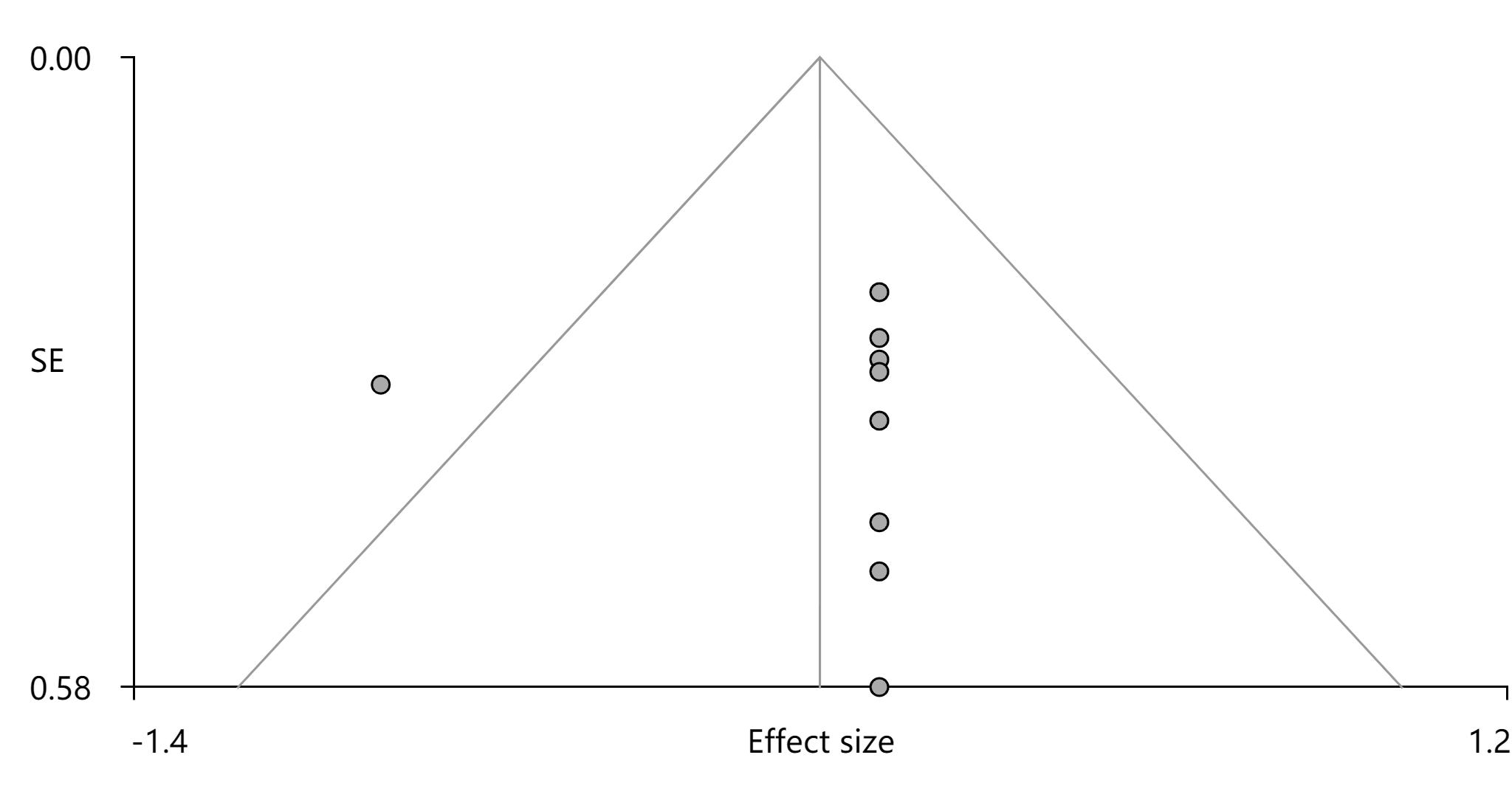


**Figure S17.** Funnel plot of cluster 9 of decreased activity in idiopathic blepharospasm.
